# Exploring causal correlations between inflammatory cytokines and systemic lupus erythematosus: A Mendelian randomization

**DOI:** 10.3389/fimmu.2022.985729

**Published:** 2023-01-19

**Authors:** Mengmeng Xiang, Yilun Wang, Zhanyan Gao, Jie Wang, Qian Chen, Zhan Sun, Jun Liang, Jinhua Xu

**Affiliations:** ^1^ Department of Dermatology, Huashan Hospital, Fudan University, Shanghai, China; ^2^ Shanghai Institute of Dermatology, Shanghai, China

**Keywords:** systemic lupus erythematosus, biomarkers, Mendelian randomization, GWAS, inflammation

## Abstract

**Objectives:**

Previous studies have reported that a few inflammatory cytokines have associations with systemic lupus erythematosus (SLE)—for example, IL-6, IL-17, and macrophage inflammatory protein (MIP). This Mendelian randomization was conducted to further assess the causal correlations between 41 inflammatory cytokines and SLE.

**Methods:**

The two-sample Mendelian randomization utilized genetic variances of SLE from a large publicly available genome-wide association study (GWAS) (7,219 cases and 15,991 controls of European ancestry) and inflammatory cytokines from a GWAS summary containing 8,293 healthy participants. Causalities of exposures and outcomes were explored mainly using inverse variance weighted method. In addition, multiple sensitivity analyses including MR-Egger, weighted median, simple mode, weighted mode, and MR-PRESSO were simultaneously applied to strengthen the final results.

**Results:**

The results indicated that cutaneous T cell-attracting chemokine (CTACK) and IL-17 may be suggestively associated with the risk of SLE (odds ratio, OR: 1.21, 95%CI: 1.04–1.41, *p* = 0.015; OR: 1.37, 95%CI: 1.03–1.82, *p* = 0.029). In addition, cytokines including beta nerve growth factor, basic fibroblast growth factor, IL-4, IL-6, interferon gamma-induced protein 10, monokine induced by interferon-gamma, MIP1b, stromal cell-derived factor-1 alpha, and tumor necrosis factor-alpha are suggested to be the consequences of SLE disease (Beta: 0.035, *p* = 0.014; Beta: 0.021, *p* = 0.032; Beta: 0.024, *p* = 0.013; Beta: 0.019, *p* = 0.042; Beta: 0.040, *p* = 0.005; Beta: 0.046, *p* = 0.001; Beta: 0.021, *p* = 0.029; Beta: 0.019, *p* = 0.045; Beta: 0.029, *p* = 0.048).

**Conclusion:**

This study suggested that CTACK and IL-17 are probably the factors correlated with SLE etiology, while a couple of inflammatory cytokines are more likely to be involved in SLE development downstream.

## Introduction

Systemic lupus erythematosus (SLE) is a chronic autoimmune disease where the immune system is dysregulated, manifesting as the deposition of immune complex in multiple organs and aberrantly the activation of innate and adaptive immunity (1). As a systemic disorder with great heterogeneity, the specific etiology and the pathophysiology of SLE have remained elusive; nonetheless, the crucial function of inflammation in disease onsets and relapses has been confirmed and validated by a number of prior research (2). The abnormal inflammatory state in SLE patients is characterized by excessive and improper functions of immune effector cells, along with elevated circulating levels of certain pro-inflammatory cytokines including IL-6, IL-1β, and TNFα, which ultimately lead to damage of the target tissue (3, 4). A few other cytokines have been reported to be closely correlated with SLE in previous studies—for example, IL-17 and macrophage inflammatory protein (MIP) (5, 6). However, it is still debatable whether systemic inflammation is a cause of SLE or it is due to disease progression, subsequent infection, and medication usage after the onset of SLE. Even while a few observational studies have attempted to elucidate the associations between inflammatory cytokines and SLE, the findings drawn from these studies may be skewed by unanticipated confounding variables or reverse causation, making definite causal correlations difficult to establish (7).

Mendelian randomization (MR) is known as the analytic approach to infer the causal effect of an exposure on an outcome using genetic variations in non-experimental data (8). Because alleles are assigned at random during meiosis, MR can reduce both conventional confounding variables and reverse causation, providing better evidence of causal inference (9). Two-sample MR analysis allows researchers to evaluate the instrument–exposure and instrument–outcome connections in two separate population samples, enhancing the test’s applicability and efficacy (10). In this study, we first extracted valid genetic variants from the published genome-wide association study (GWAS) summary data of 41 inflammatory cytokines in order to investigate their correlations with SLE, and then the direction of causation was further explored by reversing the exposure and outcome.

## Methods

This MR analysis included GWAS summary statistics that have already been published. The ethics committee at each institutional review board authorized all participants’ written informed permission in separate studies. No extra ethical approval or informed consent was required. The STROBE-MR checklist has been checked and uploaded as supplementary data (11).

### MR assumptions

**Figure 1:**
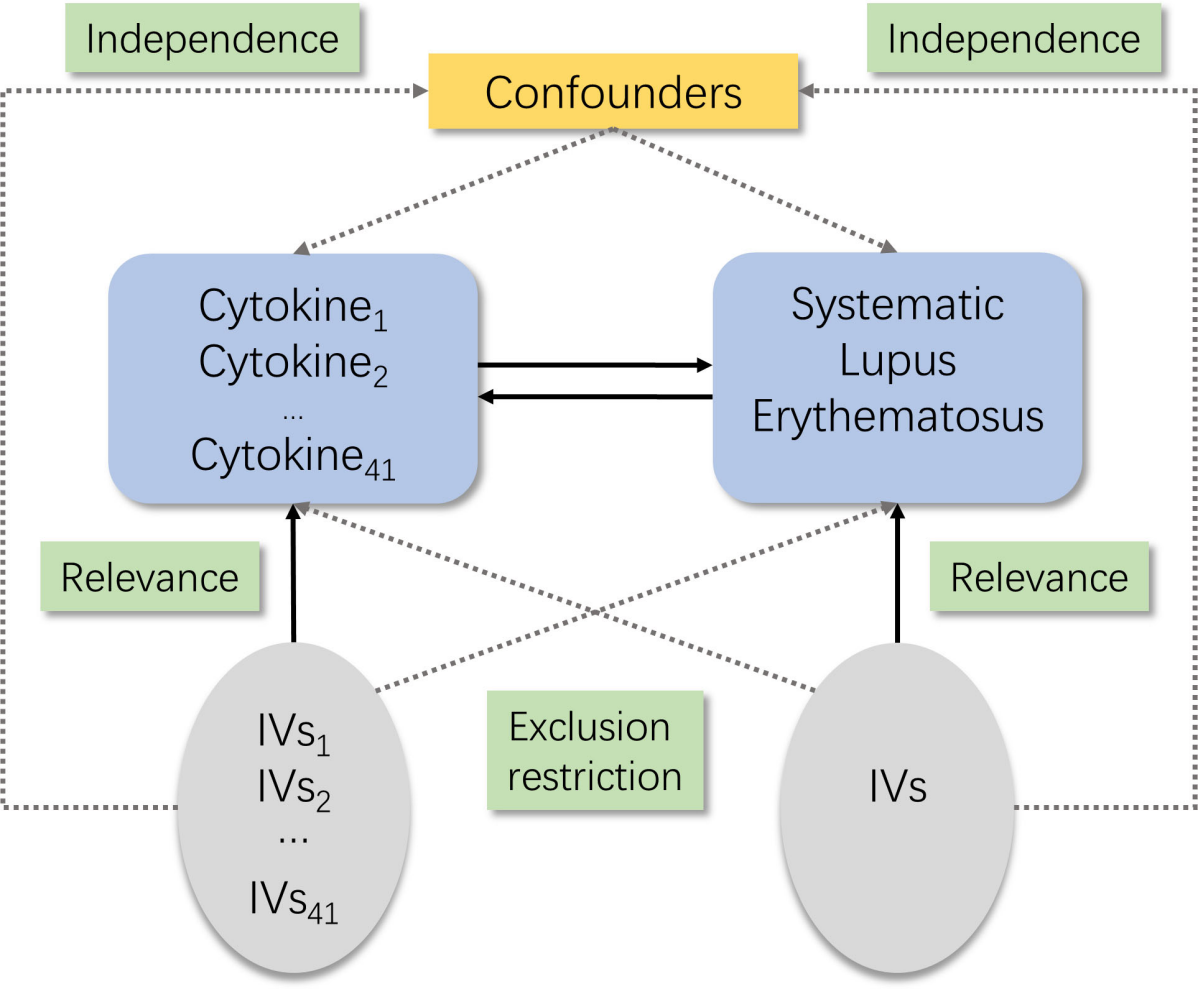
Schematic of the study design in this bidirectional Mendelian randomization (MR) analysis. Significant instrumental variables were selected for 41 inflammatory cytokines and systemic lupus erythematosus, and the bidirectional causalities were then explored. Three basic assumptions of MR analysis were illustrated in this causal directed acyclic graph, namely, relevance, independence, and exclusion restrictions.

There are three core assumptions of MR analysis, namely, relevance, independence, and exclusion restriction (12). It is assumed that the selected genetic variants are related with the risk factor (relevance) but not with any confounders in the risk factor–outcome association (independence) and that they are not connected with the outcome *via* any pathways other than the risk factor of interest (exclusion restriction). Here in this bidirectional study, two GWASs were utilized to select genetically significant SNPs for 41 inflammatory cytokines and SLE (**Figure 1**).

### Instrumental variable selection

First, we set *p <*5 × 10^-8^ as the genome-wide significant threshold to select strongly associated SNPs with SLE and inflammatory cytokines. Since very few SNPs were identified for part of cytokines when they were as the exposure, a higher cutoff (*p <*5 × 10^-6^) was chosen. Second, to avoid linkage disequilibrium, we clumped these SNPs (kb = 10,000, *r* ^2^ = 0.001). Palindromic SNPs were discarded since we could not be sure that these SNPs were aligned in the same direction for exposure and outcome in the GWASs of systemic inflammatory regulators. Third, the proportion of variance in exposure was calculated using the *R* ^2^ value of each SNP, and the instrument strength was estimated using the F-statistic to avoid weak instrument bias (13, 14). Lastly, we would substitute the SNPs that were unavailable in the outcome summary with proxy SNPs (*R* ^2^ > 0.9) from LDlink (https://ldlink.nci.nih.gov/) (15).

### Data source

Two datasets used in this MR analysis were both from publicly available summarized GWAS data. The one of SLE was obtained from a meta-analysis study comprising 7,219 cases and 15,991 controls of European ancestry (16). The SLE patients were all diagnosed according to the standard American College of Rheumatology classification criteria. The detailed study design and data control process had been reported elsewhere. For inflammatory cytokines, the data was from the study providing genome variant associations with 41 cytokines and growth factors in 8,293 Finnish individuals (17). This study combined the results from The Cardiovascular Risk in Young Finns Study (YFS) and FINRISK surveys. The average participant ages are 37 years for YFS study and 60 years for FINRISK survey. There would be no overlap in population selection between the exposure group and the outcome group.

### Statistical analysis

The causal association could be evaluated by the inverse variance weighted method (IVW). It is the most efficient method with greatest statistical power; however, it assumes that all genetic variants are valid instrumental variables, an assumption that may not hold in practice (7). Thus, other robust methods which do not require all genetic variants to be valid IVs were also employed to give consistent estimates of a causal parameter. The weighted median method has a higher tolerance of invalid IVs and can generate credible estimates when more than half of the weight corresponds to valid IVs (18). The MR-Egger approach provides consistent estimations of the causative effect under the Instrument Strength Independent of Direct Effect (InSIDE) assumption, and we evaluated the MR-Egger regression intercept as a measure of directional pleiotropy test (*p <*0.05 was judged significant) (19). However, a significant level of regression dilution still takes place when the heterogeneity *I* ^2^ statistic, measuring the strength of No Measurement Error (NOME) violation for IVs for the MR-Egger approach, is low (*I* ^2^ < 90%). The SIMEX approach can be used to fix attenuation bias when the NOME assumption is broken (18). In addition, simple mode and weighted mode analyses were also applied as the sensitivity analyses. MR-PRESSO method can identify an outlier genetic variant with horizontal pleiotropy, assuming over 50% of the instruments are valid (20). For IV exposure relevance ascertainment, F-statistic was approximated based on the summary level data. If *F >*10, the correlation was assumed to be strong enough to avoid the weak IV bias. Cochrane’s *Q*-statistic from IVW was used to measure heterogeneity among the estimates from each SNP.

The major assessment for each regulator among all these approaches listed above was chosen in accordance with the recommended strategy, which would take into account three fundamental assumptions, NOME and InSIDE. (21). If MR-PRESSO reports any outlier SNPs, the outliers will be first excluded, and the remaining IVs will then be further assessed for the suitable statistical strategy. Once the suggested approach had been decided upon, sensitivity studies for the causalities were carried out concurrently using other analytical techniques.

We adopted a Bonferroni correction due to the number of systemic inflammatory regulators examined (*p* < 0.0012, Bonferroni correction with 41 tests). Suggestive association results were those that were significant (*p* < 0.05) before but not after multiple-comparison correction (*p* < 0.0012, Bonferroni correction with 41 tests) (22). To examine the stability of effect sizes and to find the specific SNP that impacted the relationship disproportionately, a leave-one-out sensitivity analysis was performed by deleting each SNP, in turn, and applying the IVW approach to the effect of the remaining SNPs. If the SNP as IV contains missing data in the exposure or outcome summary, it would be omitted. TwoSample (23) MR package and MR-PRESSO (20) in R (version 4.1.2) were used to conduct the analysis. The study was not pre-registered at any platform.

## Results

### Influence of 41 inflammatory cytokines on SLE

Nine out of forty-one accessible systemic inflammatory regulators had three or more valid genetic variants when the cutoff value for genome-wide significance was set to 5 × 10^-8^, while for the remaining cytokines a higher threshold (*P <*5 × 10^-6^) was used to ensure adequate SNPs for further MR analysis. The variance explained by the SNPs for each inflammatory cytokine ranged from 3% to 26%, and the F-statistic values were all above 10, indicating that weak instrument bias is unlikely to be significant (**Supplementary Tables S1–S3**).

Assumption checks for determining the main analytic tools were done for all 41 regulators. Except for monocyte-specific chemokine 3 (MCP3), monokine induced by interferon gamma (MIG), and TNF-related apoptosis-inducing ligand (TRAIL), all cytokines were analyzed using the IVW methodology as the primary analytical method, with no evidence of heterogeneity and no week instruments (21). For MCP3, MR-Egger (SIMEX) was chosen as the primary approach because the *p*-values for the *Q*-tests from IVW and MR-Egger were both below 0.05, and *I* ^2^ was lower than 90%. There was no heterogeneity and no week instruments in the MIG and TRAIL when the outlier SNP reported by MR-PRESSO was removed from IVs; hence, the IVW approach was used (**Supplementary Table S2**).

**Figure 2:**
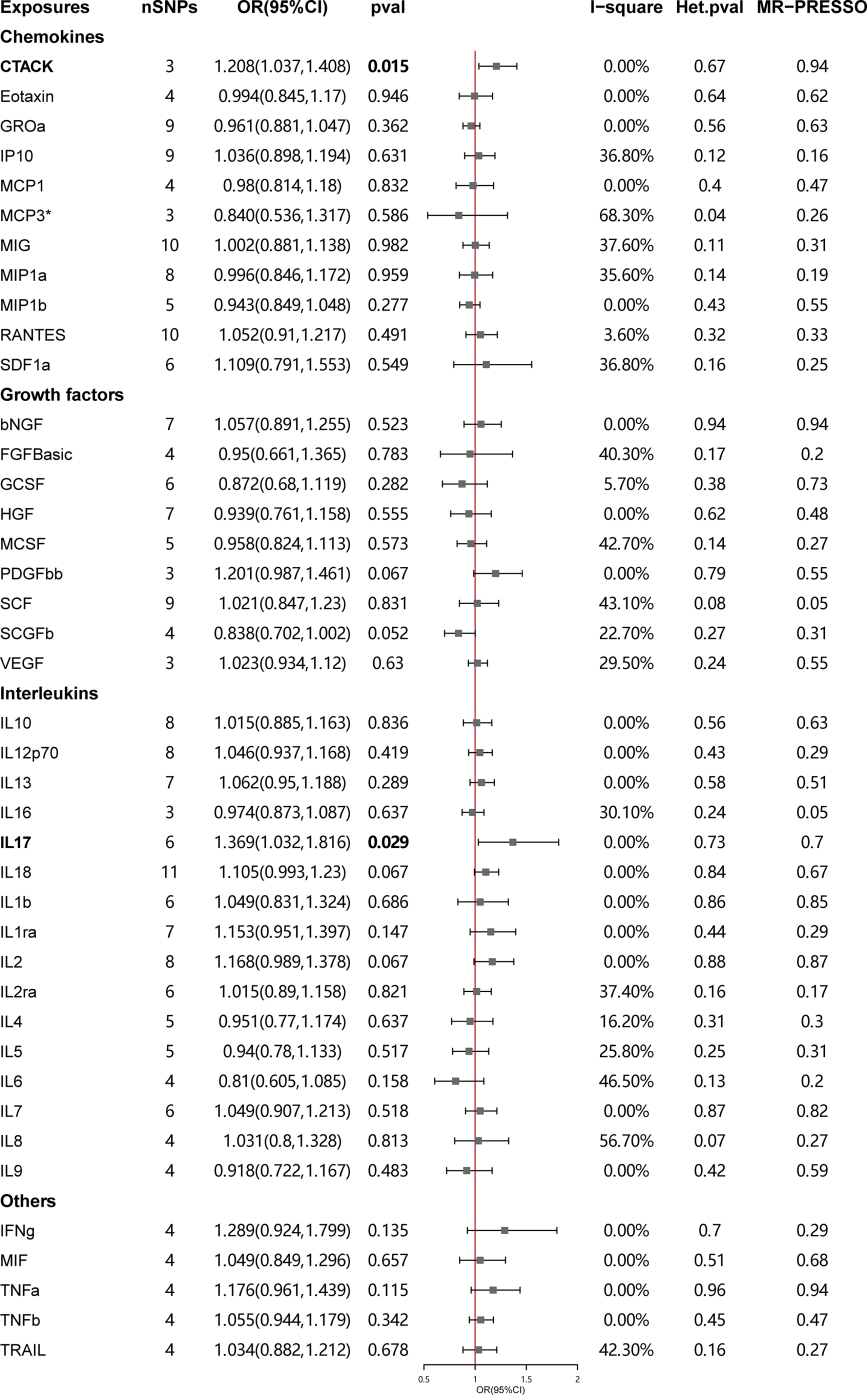
Causal correlations of 41 inflammatory cytokines on systemic lupus erythematosus. The change in the odds ratio (OR) of systemic lupus erythematosus per one-SD rise in the cytokine level is shown by OR and 95% confidence interval. *P*-value 0.05/41 = 0.0012 was found significant after multiple-comparison correction. The results from inverse variance weighted method were shown for all cytokines, except for MCP3^*^ wherein MR-Egger (SIMEX) was considered as the recommended method. bNGF, beta nerve growth factor; CTACK, cutaneous T cell-attracting chemokine; FGFBasic, basic fibroblast growth factor; GCSF, granulocyte colony-stimulating factor; GROa, growth-regulated oncogene-a; HGF, hepatocyte growth factor; IFNg, interferon gamma; IL, interleukin; IP, interferon gamma-induced protein 10; MCP1, monocyte chemotactic protein 1; MCP3, monocyte-specific chemokine 3; MCSF, macrophage colony-stimulating factor; MIF, macrophage migration inhibitory factor; MIG, monokine induced by interferon gamma; MIP1a, macrophage inflammatory protein-1a; MIP1b, macrophage inflammatory protein-1b; PDGFbb, platelet-derived growth factor BB; RANTES, regulated upon activation normal T cell expressed and secreted factor; SCF, stem cell factor; SCGFb, stem cell growth factor beta; SDF1a, stromal cell-derived factor-1 alpha; SNPs, single-nucleotide polymorphisms; TNFa, tumor necrosis factor alpha; TNFb, tumor necrosis factor beta; TRAIL, TNF-related apoptosis-inducing ligand; VEGF, vascular endothelial growth factor.

**Figure 3:**
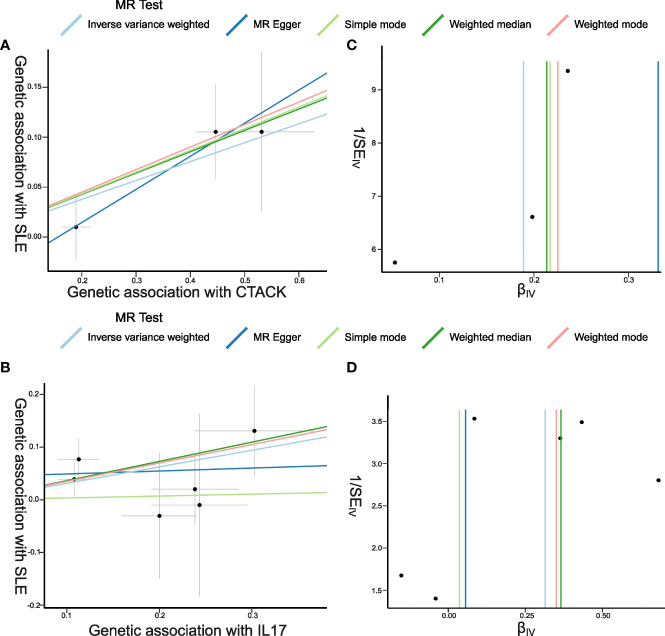
Scatter plots and funnel plots of Mendelian randomization (MR) analyses for CTACK and IL-17 in systemic lupus erythematosus (SLE). **(A, B)** Individual inverse variance (IV) associations with cytokine risk are displayed *versus* individual IV associations with SLE in black dots. The 95%CI of odd ratio for each IV is shown by vertical and horizontal lines. The slope of the lines represents the estimated causal effect of the MR methods. **(C, D)** The funnel plots show the inverse variance weighted MR estimate of each cytokine single-nucleotide polymorphism with SLE *versus* 1/standard error (1/SE_IV_).

The primary results of the main MR analyses for the 41 cytokines are presented in **Figure 2** and **Supplementary Table S1**. It was found by the IVW method that genetically determined higher T cell-attracting chemokine (CTACK) levels (one-SD increase) were suggestively associated with 21% higher odds for SLE (OR: 1.21, 95%CI: 1.04–1.41, *p* = 0.015). It was in accordance with the weighted median method (OR: 1.24, 95%CI: 1.05–1.46, *p* = 0.011). The MR Egger analysis failed to detect a statistically significant association but indicated a similar changing trend (OR: 1.39, 95%CI: 0.97–2.00, *p* = 0.33). The same causal relationship was observed in IL-17 with SLE (IVW—OR: 1.37, 95%CI: 1.03–1.82, *p* = 0.029; weighted median—OR: 1.44, 95%CI: 1.00–2.07, *p* = 0.049). The scatter plots and funnel plots of Mendelian randomization analyses for CTACK and IL-17 in SLE are exhibited in **Figure 3**.

There was no evidence for heterogeneity in the associations of CTACK and IL-17 as measured by *I* ^2^ and Cochrane’s *Q* (*I* ^2^: 0%; *p* = 0.67; *I* ^2^: 0%; *p* = 0.73), and no outlier SNPs were detected with the MR-PRESSO method. Furthermore, the MR-Egger intercept revealed no evidence of directional pleiotropy effects (*p* = 0.55; *p* = 0.48) (**Figures 2**, **3**; **Supplementary Table S2**). Details of the SNPs are presented in **Supplementary Table S3**. The forest plots and leave-one-out sensitivity analyses of Mendelian randomization analyses for CTACK and IL-17 in SLE are shown in **Supplementary Figure S1**.

### Influence of SLE on 41 inflammatory cytokines

Forty significant SNPs were extracted as the IVs for SLE. When the inflammatory cytokines were viewed as the outcomes, two and eight SNPs were not accessible in the result GWAS for MCP3 and tumor necrosis factor beta correspondingly, with no proxy SNPs available, thus leaving 38 and 32 SNPs to be included in the following study. One SNP (rs12524498) for vascular endothelial growth factor (VEGF) could not be found, so the proxy (rs115751548) was used instead. The median value of *F*-statistic was 47.87 (range, 29.73–461) (**Supplementary Tables S4–S6**).

As no heterogeneity and weak instruments were observed, the IVW technique was used as the primary evaluation of corresponding casualties with SLE for 37 regulators. Outlier SNPs for growth-regulated oncogene-a, interferon gamma-induced protein 10 (IP10), and MIG were reported utilizing MR-PRESSO test. The heterogeneity for three cytokines became insignificant once the outliers were removed (*I* ^2^: 15.1%; Cochrane’s *Q p* = 0.21; *I* ^2^: 12.8%; Cochrane’s *Q p* = 0.25; *I* ^2^: 0%; Cochrane’s *Q p* = 0.48), so IVW was also applicable as the main test. For MCP1, MR-Egger (SIMEX) was implemented as the concluding results with heterogeneity existence, *I* ^2^ <90% and InSIDE assumption fulfillment (21) (**Supplementary Table S5**).

**Figure 4:**
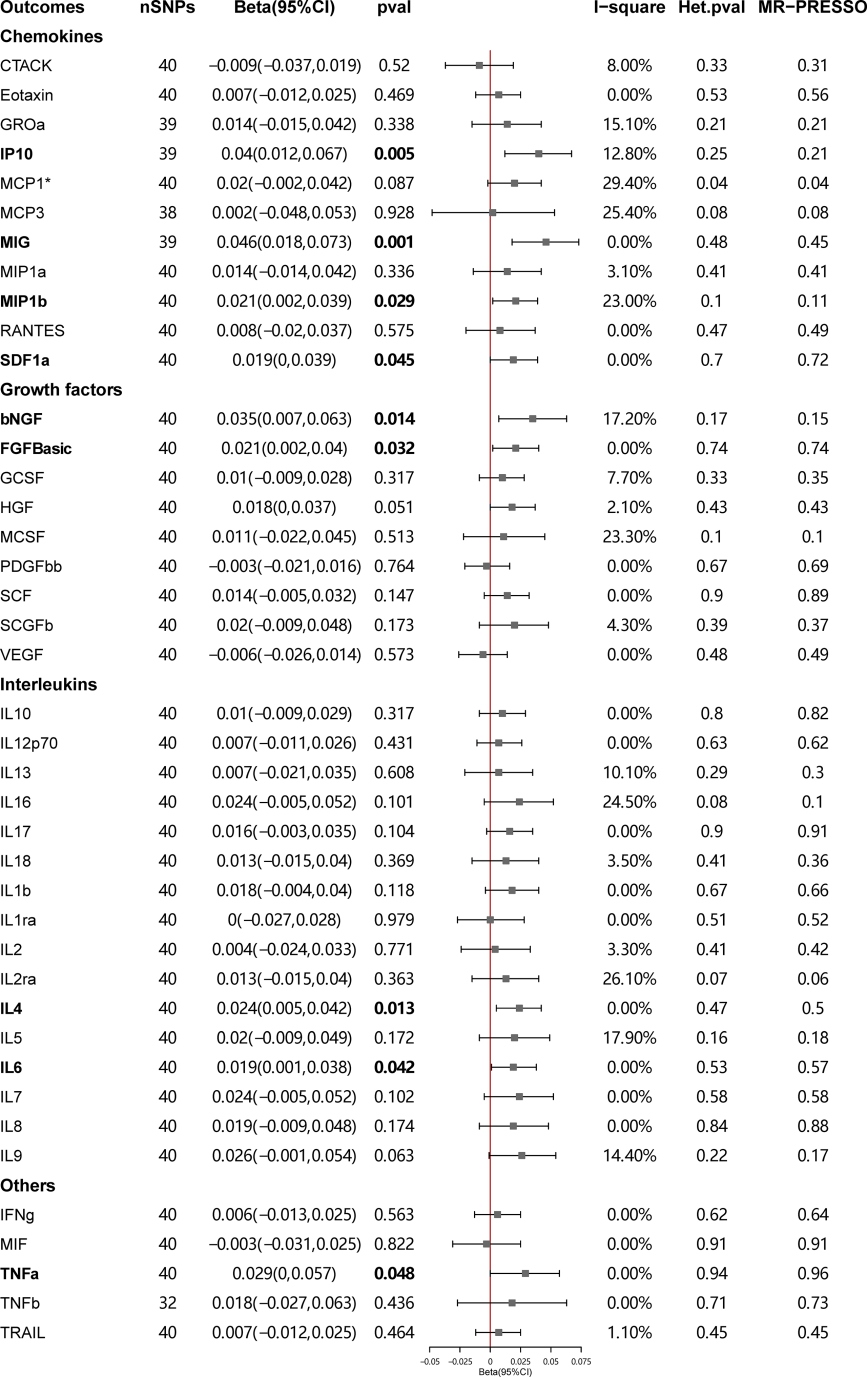
Causal correlations of systemic lupus erythematosus (SLE) on 41 inflammatory cytokines. The change in the SD of inflammatory cytokines per log odds increase in SLE is represented by beta and the 95% confidence interval. *P*-value 0.05/41 = 0.0012 was found significant after multiple-comparison correction. The results from inverse variance weighted method were shown for all cytokines, except for MCP1^*^ wherein MR-Egger (SIMEX) was considered as the recommended method. bNGF, beta nerve growth factor; CTACK, cutaneous T cell-attracting chemokine; FGFBasic, basic fibroblast growth factor; GCSF, granulocyte colony-stimulating factor; GROssa, growth-regulated oncogene-a; HGF, hepatocyte growth factor; IFNg, interferon gamma; IL, interleukin; IP, interferon gamma induced protein 10; MCP1, monocyte chemotactic protein 1; MCP3, monocyte-specific chemokine 3; MCSF, macrophage colony-stimulating factor; MIF, macrophage migration inhibitory factor; MIG, monokine induced by interferon gamma; MIP1a, macrophage inflammatory protein-1a; MIP1b, macrophage inflammatory protein-1b; PDGFbb, platelet-derived growth factor BB; RANTES, regulated upon activation normal T cell expressed and secreted factor; SCF, stem cell factor; SCGFb, stem cell growth factor beta; SDF1a, stromal cell-derived factor-1 alpha; SNPs, single-nucleotide polymorphisms; TNFa, tumor necrosis factor alpha; TNFb, tumor necrosis factor beta; TRAIL, TNF-related apoptosis-inducing ligand; VEGF, vascular endothelial growth factor.

**Figure 4** presents the outcomes from the MR analysis regarding the causality between SLE and inflammatory cytokines. The findings of the IVW method revealed that SLE was suggestively correlated with an elevated level of beta nerve growth factor (bNGF) (Beta: 0.035, 95%CI: 0.007–0.063, *p* = 0.014), basic fibroblast growth factor (FGFbasic) (Beta: 0.021, 95%CI: 0.002–0.04, *p* = 0.032), interleukin-4 (IL-4) (Beta: 0.024, 95%CI: 0.005–0.042, *p* = 0.013), IL-6 (Beta: 0.019, 95%CI: 0.001–0.038, *p* = 0.042), IP10 (Beta: 0.040, 95%CI: 0.012–0.067, *p* = 0.005), MIG (Beta: 0.046, 95%CI: 0.018–0.073, *p* = 0.001), macrophage inflammatory protein-1b (MIP1b) (Beta: 0.021, 95%CI: 0.002–0.039, *p* = 0.029), stromal cell-derived factor-1 alpha (SDF1a) (Beta: 0.019, 95%CI: 0.00–0.039, *p* = 0.045), and tumor necrosis factor alpha (TNFa) (Beta: 0.029, 95%CI: 0–0.057, *p* = 0.048).

**Figure 5:**
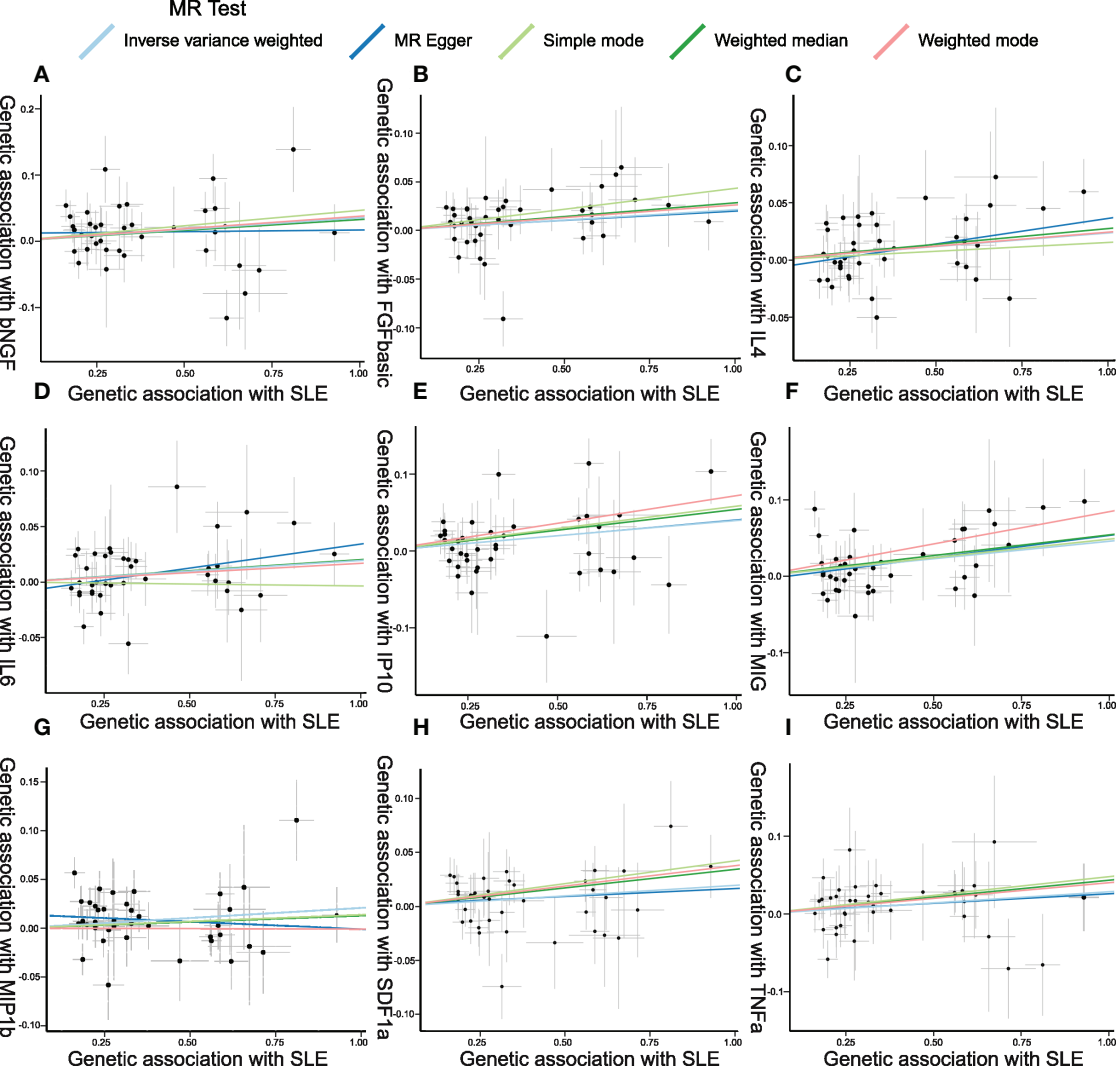
Scatter plots of Mendelian randomization (MR) analyses between systemic lupus erythematosus (SLE) and inflammatory cytokines. Individual inverse variance (IV) associations with SLE risk are displayed *versus* individual IV associations with cytokines in black dots. The 95%CI of the odds ratio for each IV is shown by the vertical and horizontal lines. The slope of the lines represents the estimated causal effect of the MR methods. **(A–I)**: bNGF, FGFbasic, IL-4, IL-6, IP10, MIG, MIP1b, SDF1a, and TNFa.

**Figure 6:**
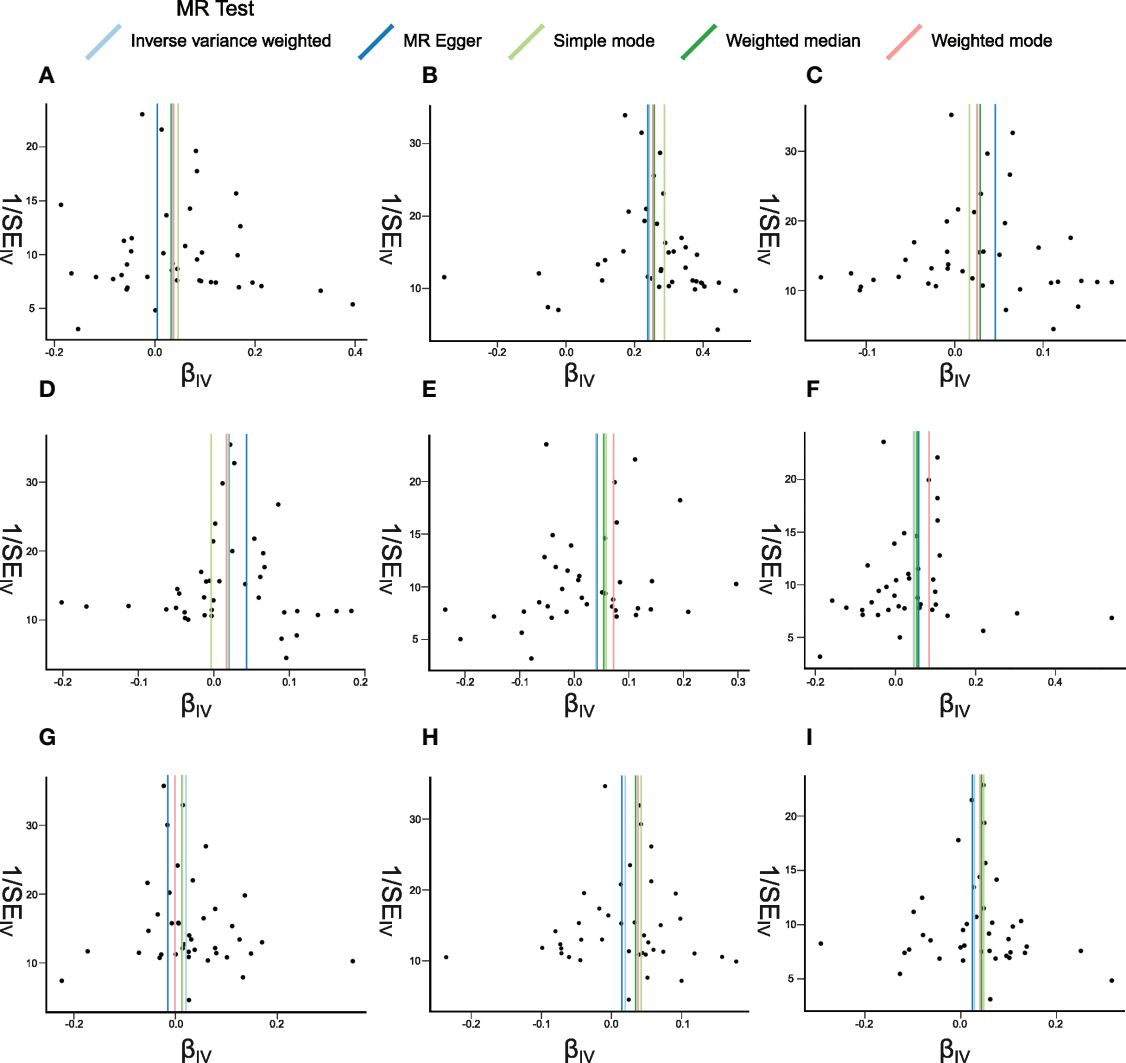
Funnel plots of Mendelian randomization (MR) analyses between systemic lupus erythematosus (SLE) and inflammatory cytokines. The funnel plots show the inverse variance weighted MR estimate of each SLE single-nucleotide polymorphism with cytokines *versus* 1/standard error (1/SE_IV_). **(A–I)** bNGF, FGFbasic, IL-4, IL-6, IP10, MIG, MIP1b, SDF1a, and TNFa.

The weighted median estimator provided estimates of the same magnitude as the IVW analysis for IL-4 (Beta: 0.028, 95%CI: 0–0.055, *p* = 0.046), IP10 (Beta: 0.054, 95%CI: 0.009–0.099, *p* = 0.019), MIG (Beta: 0.054, 95%CI: 0.013–0.095, *p* = 0.010), SDF1a (Beta: 0.034, 95%CI: 0.006–0.063, *p* = 0.019), and TNFa (Beta: 0.043, 95%CI: 0.005–0.082, *p* = 0.026). In addition, the associations assessed by MR-Egger regression analysis were similar with IVW for IL-4 (Beta: 0.045 95%CI: 0.004–0.085, *p* = 0.036) and IL-6 (Beta: 0.043, 95%CI: 0.003–0.083, *p* = 0.041) (**Figure 5**; **Supplementary Table S4**). The funnel plots of suggestively correlated cytokines are shown in **Figure 6**, while the forest plots and leave-one-out sensitivity analyses of all suggestively significant regulators are presented in **Supplementary Figures S2, S3**.

## Discussion

In this two-sample MR analysis, we first investigated the causative relationships when 41 biomarkers, including growth factors, interleukins, and chemokines, were seen as exposures and SLE as the outcome, and it was shown that CTACK and IL-17 may suggestively be the upstream causes of SLE. Furthermore, if SLE was defined as the exposure variable in the MR, it could suggestively lead to a rise in bNGF, FGFbasic, IP10, IL-4, IL-6, MIG, MIP1b, SDF1a, and TNFa levels *via* causative pathways. There were no reverse causalities found between a single biomarker and SLE. It can be concluded that several biomarkers may act as the initiatives in the onset of SLE, while a couple of other inflammatory regulators are more likely to lie downstream during disease progression.

Until now, extensive research exploring the correlations between SLE and a wide spectrum of inflammation-related cytokines has been published—for example, the meta-analyses had reported that VEGF, IL-18, and IL-17 were elevated in patients with SLE than in healthy participants, and they could even serve as indicator of disease activity and severity (24–26). In SLE patients, a specific profile of cytokine Th17/Treg imbalance was observed, which was characterized by a decline in IFN-γ and TGF-β1 with a rise in IL-6 and IL-17 levels (4). Critical immune cells that can produce regulatory or inflammatory cytokines like B cells, subsets of T cells, and macrophages were also unveiled to display aberrant functions in SLE (27). However, even with abundant results from observational studies, the casual correlations of SLE with important cytokines still cannot be established due to the limitations of classical epidemiology (28). The interpretation of a connection between an exposure and a disease outcome in observational data as a causal relationship depends on untestable and mostly unachievable assumptions such the absence of unexpected confounding and reverse causation (29). Herein the increased levels of inflammatory cytokines in SLE patients may indicate the cause of disease, a side effect of treatment, an underlying infection state, or other concurrent pathological immune responses which could not be distinguished by ordinary observational studies.

The main conclusion drawn from this MR research is that CTACK and IL-17 may suggestively have a role in the beginning of SLE. CTACK is a kind of skin-specific chemokines expressed by keratinocytes, which can mediate the migration of lymphocytes into the skin by binding to CCR10 (30). It had been thoroughly researched in skin diseases including atopic dermatitis (AD) and psoriasis (31, 32). Serum CTACK was shown to be higher in AD patients and correlated with disease severity both in children with atopic and non-atopic dermatitis as well as children with asthma and urticaria (33, 34). The expression of CTACK would be upregulated with the stimulation of TNF-α and IL-1β and be reduced cutaneously during the administration of etanercept in psoriasis patients (35, 36). Even though there have been few studies on CTACK in lupus, its underlying function in the formation of lupus skin lesions should be investigated, and exploratory research utilizing more comprehensive data should be conducted to elucidate the link between CTACK levels and SLE.

In the case of IL-17, which is a novel therapeutic target in SLE, various studies have confirmed its significance in the disease’s etiology (37). IL-17, mainly produced by Th17 cells, can promote the recruitment of inflammatory cells, amplify the production of inflammatory cytokines and chemokines, and activate other cells like keratinocytes and macrophages (38). In addition, the imbalance between Th17 and Treg cells, as well as IL-17-related cytokine-driven inflammation, is widely recognized to have a role in autoantibody generation and organ damage in SLE (39). More research should be performed on the prognostic and diagnostic value of circulating IL-17, its involvement in pathogenesis, and the efficacy of IL-17-targeted treatment methods in SLE based on the findings of previous studies and this MR study. There could potentially exist an interplay between IL-17 and CTACK, although few studies had reported on this, and how the two cytokines function in the immune initiation and set off the subsequent chain of events in the disease context remain as meaningful questions to be solved.

Prior research had revealed that several inflammatory regulators, such as IL-4, IL-6, TNF-α, IP10, and MIG, had a tight link with SLE, which supported our findings (40–42). It was found that the secreted plasma levels of IL-4 and IL-6, which are both Th2 cytokines, were elevated in SLE patients, suggesting a distinct plasma cytokine profile (4). Similarly for TNF-α, a rise in the plasma levels of patients was noted, and it can be one of the strongest markers for differentiating between patients and controls, particularly those with renal involvement (43, 44). IP10 and MIG are both proinflammatory Th1 chemokines that can engage in the regulation of various immune responses (45, 46). Their plasma levels were higher in SLE patients and positively correlated with SLEDAI score (47).

However, the majority of previous investigations focused on the variations in the cytokine production levels between SLE patients and healthy controls, and how these cytokines change led to other pathological alterations (48). Additionally, several exploratory trials that sought to treat a small cohort of SLE patients by targeting these cytokines failed to provide a satisfactory therapeutic effect, and no drugs targeting these immune regulators have been approved for the treatment of SLE so far. The clinical trials of IL-6 monoclonal antibody and IL-6 receptor antagonist had indicated that such treatment showed various effectiveness with great heterogeneity, and there was still a long way to go before they could be applied in clinical settings in the future (49, 50). For treatment blocking TNF-α in SLE, the risks outweighed the benefits, and it may only be beneficial in a limited subset of patients (51). The underlying reasons for these unsatisfactory treatment outcomes have remained unclear, while our study implied that these cytokines were more likely to be the downstream consequences of SLE progression so that the corresponding treatment may fail to block disease development from the very beginning. If the immune regulators exhibit specific exposure–outcome linkages with disease onset, their therapeutic utility may be more plausible.

This is the first Mendelian randomization research to evaluate the causal relationship between SLE and 41 inflammatory cytokines. However, there are several limitations that must be considered. First, due to the limitations of the MR analysis, the second and third assumptions could not be properly examined, potentially resulting in bias. Second, the data for our investigation came from two large-scale GWASs, and subgroup analyses were unachievable due to a lack of specific demographic information and clinical records of study patients. Third, ethnic bias may be present because the study individuals were of European descent, so it should be taken with caution if the conclusions would be applied in other races. More research should be conducted to confirm our results and to try to apply them to clinical diagnosis procedures and therapy options.

## Data availability statement

The datasets presented in this study can be found in online repositories. The names of the repository/repositories and accession number(s) can be found in the article/**Supplementary Material**.

## Author contributions

MX and YW designed the study and edited the manuscript. ZG, ZS, and JW performed the statistical analysis and drafted the manuscript. QC, JX, JL and ZS reviewed and edited the manuscript. All authors contributed to the article and approved the submitted version.

## References

[1] Koutsokeras T Healy T. Systemic lupus erythematosus and lupus nephritis. Nat Rev Drug Discov (2014) 13:173–4. doi: 10.1038/nrd4227 24525782

[2] Rigante D Esposito S. Infections and systemic lupus erythematosus: Binding or sparring partners? Int J Mol Sci (2015) 16:17331–43. doi: 10.3390/ijms160817331 PMC4581196 26230690

[3] Weinstein A Alexander RV Zack DJ. A review of complement activation in SLE. Curr Rheumatol Rep (2021) 23:16. doi: 10.1007/s11926-021-00984-1 33569681 PMC7875837

[4] Talaat RM Mohamed SF Bassyouni IH Raouf AA. Th1/Th2/Th17/Treg cytokine imbalance in systemic lupus erythematosus (SLE) patients: Correlation with disease activity. Cytokine (2015) 72:146–53. doi: 10.1016/j.cyto.2014.12.027 25647269

[5] Thanou A Jupe E Purushothaman M Niewold TB Munroe ME. Clinical disease activity and flare in SLE: Current concepts and novel biomarkers. J Autoimmun (2021) 119:102615. doi: 10.1016/j.jaut.2021.102615 33631651 PMC8044029

[6] Munroe ME Vista ES Guthridge JM Thompson LF Merrill JT James JA. Proinflammatory adaptive cytokine and shed tumor necrosis factor receptor levels are elevated preceding systemic lupus erythematosus disease flare: Altered inflammatory mediators preceding SLE flare. Arthritis Rheumatol (2014) 66:1888–99. doi: 10.1002/art.38573 PMC4128244 24578190

[7] Burgess S Butterworth A Thompson SG. Mendelian randomization analysis with multiple genetic variants using summarized data. Genet Epidemiol (2013) 37:658–65. doi: 10.1002/gepi.21758 PMC4377079 24114802

[8] Wootton RE Richmond RC Stuijfzand BG Lawn RB Sallis HM Taylor GMJ. Evidence for causal effects of lifetime smoking on risk for depression and schizophrenia: a mendelian randomisation study. Psychol Med (2020) 50:2435–43. doi: 10.1017/S0033291719002678 PMC7610182 31689377

[9] Burgess S Scott RA Timpson NJ Davey Smith G Thompson SG EPIC- InterAct Consortium. Using published data in mendelian randomization: a blueprint for efficient identification of causal risk factors. Eur J Epidemiol (2015) 30:543–52. doi: 10.1007/s10654-015-0011-z PMC4516908 25773750

[10] Hartwig FP Davies NM Hemani G Davey Smith G. Two-sample mendelian randomization: avoiding the downsides of a powerful, widely applicable but potentially fallible technique. Int J Epidemiol (2016) 45:1717–26. doi: 10.1093/ije/dyx028 PMC5722032 28338968

[11] Skrivankova VW Richmond RC Woolf BAR Yarmolinsky J Davies NM Swanson SA. Strengthening the reporting of observational studies in epidemiology using mendelian randomization: The STROBE-MR statement. JAMA (2021) 326:1614. doi: 10.1001/jama.2021.18236 34698778

[12] Emdin CA Khera AV Kathiresan S. Mendelian randomization. JAMA (2017) 318:1925–6. doi: 10.1001/jama.2017.17219 29164242

[13] Pierce BL Ahsan H VanderWeele TJ. Power and instrument strength requirements for mendelian randomization studies using multiple genetic variants. Int J Epidemiol (2011) 40:740–52. doi: 10.1093/ije/dyq151 PMC3147064 20813862

[14] Palmer TM Lawlor DA Harbord RM Sheehan NA Tobias JH Timpson NJ. Using multiple genetic variants as instrumental variables for modifiable risk factors. Stat Methods Med Res (2012) 21:223–42. doi: 10.1177/0962280210394459 PMC3917707 21216802

[15] Machiela MJ Chanock SJ. LDlink: a web-based application for exploring population-specific haplotype structure and linking correlated alleles of possible functional variants. Bioinformatics (2015) 31:3555–7. doi: 10.1093/bioinformatics/btv402 PMC4626747 26139635

[16] Bentham J Morris DL Cunninghame Graham DS Pinder CL Tombleson P Behrens TW. Genetic association analyses implicate aberrant regulation of innate and adaptive immunity genes in the pathogenesis of systemic lupus erythematosus. Nat Genet (2015) 47:1457–64. doi: 10.1038/ng.3434 PMC4668589 26502338

[17] Ahola-Olli AV Würtz P Havulinna AS Aalto K Pitkänen N Lehtimäki T. Genome-wide association study identifies 27 loci influencing concentrations of circulating cytokines and growth factors. Am J Hum Genet (2017) 100:40–50. doi: 10.1016/j.ajhg.2016.11.007 27989323 PMC5223028

[18] Bowden J Davey Smith G Haycock PC Burgess S. Consistent estimation in mendelian randomization with some invalid instruments using a weighted median estimator. Genet Epidemiol (2016) 40:304–14. doi: 10.1002/gepi.21965 PMC4849733 27061298

[19] Bowden J Davey Smith G Burgess S. Mendelian randomization with invalid instruments: effect estimation and bias detection through egger regression. Int J Epidemiol (2015) 44:512–25. doi: 10.1093/ije/dyv080 PMC4469799 26050253

[20] Verbanck M Chen C-Y Neale B Do R. Detection of widespread horizontal pleiotropy in causal relationships inferred from mendelian randomization between complex traits and diseases. Nat Genet (2018) 50:693–8. doi: 10.1038/s41588-018-0099-7 PMC6083837 29686387

[21] Jin H Lee S Won S. Causal evaluation of laboratory markers in type 2 diabetes on cancer and vascular diseases using various mendelian randomization tools. Front Genet (2020) 11:597420. doi: 10.3389/fgene.2020.597420 33408737 PMC7780896

[22] Georgakis MK Gill D Rannikmäe K Traylor M Anderson CD MEGASTROKE consortium of the International Stroke Genetics Consortium (ISGC). Genetically determined levels of circulating cytokines and risk of stroke: Role of monocyte chemoattractant protein-1. Circulation (2019) 139:256–68. doi: 10.1161/CIRCULATIONAHA.118.035905 PMC7477819 30586705

[23] Hemani G Zheng J Elsworth B Wade KH Haberland V Baird D. The MR-base platform supports systematic causal inference across the human phenome. eLife (2018) 7:e34408. doi: 10.7554/eLife.34408 29846171 PMC5976434

[24] Zhan H Li H Liu C Cheng L Yan S Li Y. Association of circulating vascular endothelial growth factor levels with autoimmune diseases: A systematic review and meta-analysis. Front Immunol (2021) 12:674343. doi: 10.3389/fimmu.2021.674343 34122433 PMC8191579

[25] Xiang M Feng Y Wang Y Wang J Zhang Z Liang J. Correlation between circulating interleukin-18 level and systemic lupus erythematosus: a meta-analysis. Sci Rep (2021) 11:4707. doi: 10.1038/s41598-021-84170-4 33633218 PMC7907126

[26] Shen H-H Fan Y Wang Y-N Zhao C-N Zhang Z-K Pan H-F. Elevated circulating interleukin-17 levels in patients with systemic lupus erythematosus: A meta-analysis. Immunol Invest (2020) 49:662–75. doi: 10.1080/08820139.2019.1699107 31847623

[27] Ahamada MM Jia Y Wu X. Macrophage polarization and plasticity in systemic lupus erythematosus. Front Immunol (2021) 12:734008. doi: 10.3389/fimmu.2021.734008 34987500 PMC8721097

[28] Davey Smith G Hemani G. Mendelian randomization: genetic anchors for causal inference in epidemiological studies. Hum Mol Genet (2014) 23:R89–98. doi: 10.1093/hmg/ddu328 PMC4170722 25064373

[29] Sekula P Del Greco MF Pattaro C Köttgen A. Mendelian randomization as an approach to assess causality using observational data. J Am Soc Nephrol (2016) 27:3253–65. doi: 10.1681/ASN.2016010098 PMC5084898 27486138

[30] Kunkel EJ Butcher EC. Chemokines and the tissue-specific migration of lymphocytes. Immunity (2002) 16:1–4. doi: 10.1016/S1074-7613(01)00261-8 11825560

[31] Karakawa M Kishimoto M Ohtsuki M Komine M. Calcipotriol induces the production of CTACK/CCL27, one of the potential suppressive factors in psoriasis inflammation. J Dermatol (2021) 48:1949–50. doi: 10.1111/1346-8138.16152 34505709

[32] Renert-Yuval Y Thyssen JP Bissonnette R Bieber T Kabashima K Hijnen D. Biomarkers in atopic dermatitis–a review on behalf of the international eczema council. J Allergy Clin Immunol (2021) 147:1174–1190.e1. doi: 10.1016/j.jaci.2021.01.013 33516871 PMC11304440

[33] Machura E Rusek-Zychma M Jachimowicz M Wrzask M Mazur B Kasperska-Zajac A. Serum TARC and CTACK concentrations in children with atopic dermatitis, allergic asthma, and urticaria: TARC, CTACK in allergy in children. Pediatr Allergy Immunol (2012) 23:278–84. doi: 10.1111/j.1399-3038.2011.01225.x 22017510

[34] Song TW Sohn MH Kim ES Kim KW Kim K-E. Increased serum thymus and activation-regulated chemokine and cutaneous T cell-attracting chemokine levels in children with atopic dermatitis. Clin Exp Allergy (2006) 36:346–51. doi: 10.1111/j.1365-2222.2006.02430.x 16499646

[35] Campanati A Goteri G Simonetti O Ganzetti G Giuliodori K Stramazzotti D. CTACK/CCL27 expression in psoriatic skin and its modification after administration of etanercept. Br J Dermatol (2007) 157:1155–60. doi: 10.1111/j.1365-2133.2007.08200.x 17916208

[36] Kakinuma T Saeki H Tsunemi Y Fujita H Asano N Mitsui H. Increased serum cutaneous T cell-attracting chemokine (CCL27) levels in patients with atopic dermatitis and psoriasis vulgaris. J Allergy Clin Immunol (2003) 111:592–7. doi: 10.1067/mai.2003.114 12642842

[37] McGeachy MJ Cua DJ Gaffen SL. The IL-17 family of cytokines in health and disease. Immunity (2019) 50:892–906. doi: 10.1016/j.immuni.2019.03.021 30995505 PMC6474359

[38] Ouyang W Kolls JK Zheng Y. The biological functions of T helper 17 cell effector cytokines in inflammation. Immunity (2008) 28:454–67. doi: 10.1016/j.immuni.2008.03.004 PMC3424508 18400188

[39] Koga T Ichinose K Kawakami A Tsokos GC. Current insights and future prospects for targeting IL-17 to treat patients with systemic lupus erythematosus. Front Immunol (2021) 11:624971. doi: 10.3389/fimmu.2020.624971 33597953 PMC7882681

[40] Dominguez-Gutierrez PR Ceribelli A Satoh M Sobel ES Reeves WH Chan EK. Reduced levels of CCL2 and CXCL10 in systemic lupus erythematosus patients under treatment with prednisone, mycophenolate mofetil, or hydroxychloroquine, except in a high STAT1 subset. Arthritis Res Ther (2014) 16:R23. doi: 10.1186/ar4451 24460726 PMC3978465

[41] Dong C Fu T Ji J Li Z Gu Z. The role of interleukin-4 in rheumatic diseases. Clin Exp Pharmacol Physiol (2018) 45:747–54. doi: 10.1111/1440-1681.12946 29655253

[42] Stanley S Mok CC Vanarsa K Habazi D Li J Pedroza C. Identification of low-abundance urinary biomarkers in lupus nephritis using electrochemiluminescence immunoassays. Arthritis Rheumatol (2019) 71:744–55. doi: 10.1002/art.40813 30618193

[43] Quan W An J Li G Qian G Jin M Feng C. Th Cytokine profile in childhood-onset systemic lupus erythematosus. BMC Pediatr (2021) 21:187. doi: 10.1186/s12887-021-02659-3 33882880 PMC8059275

[44] Idborg H Eketjäll S Pettersson S Gustafsson JT Zickert A Kvarnström M. TNF-α and plasma albumin as biomarkers of disease activity in systemic lupus erythematosus. Lupus Sci Med (2018) 5:e000260. doi: 10.1136/lupus-2018-000260 29955370 PMC6018889

[45] Lee EY Lee Z-H Song YW. CXCL10 and autoimmune diseases. Autoimmun Rev (2009) 8:379–83. doi: 10.1016/j.autrev.2008.12.002 19105984

[46] Lit LCW Wong CK Tam LS Li EKM Lam CWK. Raised plasma concentration and ex vivo production of inflammatory chemokines in patients with systemic lupus erythematosus. Ann Rheum Dis (2006) 65:209–15. doi: 10.1136/ard.2005.038315 PMC1798029 15975968

[47] El-Gohary A Hegazy A Abbas M Kamel N Nasef SI. Serum and urinary interferon-Gamma-Inducible protein 10 in lupus nephritis. J Clin Lab Anal (2016) 30:1135–8. doi: 10.1002/jcla.21993 PMC6806674 27184880

[48] Rönnblom L Elkon KB. Cytokines as therapeutic targets in SLE. Nat Rev Rheumatol (2010) 6:339–47. doi: 10.1038/nrrheum.2010.64 20440285

[49] Wallace DJ Strand V Merrill JT Popa S Spindler AJ Eimon A. Efficacy and safety of an interleukin 6 monoclonal antibody for the treatment of systemic lupus erythematosus: a phase II dose-ranging randomised controlled trial. Ann Rheum Dis (2017) 76:534–42. doi: 10.1136/annrheumdis-2016-209668 PMC5446001 27672124

[50] Illei GG Shirota Y Yarboro CH Daruwalla J Tackey E Takada K. Tocilizumab in systemic lupus erythematosus: Data on safety, preliminary efficacy, and impact on circulating plasma cells from an open-label phase I dosage-escalation study. Arthritis Rheum (2010) 62:542–52. doi: 10.1002/art.27221 PMC3057537 20112381

[51] Idborg H Oke V. Cytokines as biomarkers in systemic lupus erythematosus: Value for diagnosis and drug therapy. Int J Mol Sci (2021) 22:11327. doi: 10.3390/ijms222111327 34768756 PMC8582965

