# Supplementary material for: Exploring causal correlations between inflammatory cytokines and systemic lupus erythematosus: A Mendelian randomization
Source: Front Immunol. 2023 Jan 19;13:985729. doi: 10.3389/fimmu.2022.985729 (PMC9893779; doi:10.3389/fimmu.2022.985729)
Supplement: Supplementary file 1 [file DataSheet_1.pdf]

Table S1. MR estimates of forty-one inflammatory cytokines on SLE.

			Inverse variance weighted			MR-Egger			Weighted Median			Simple Mode			Weighted Mode		
Category	Exposures	No. of SNPs	OR	95% CI	pval	OR	95% CI	pval	OR	95% CI	pval	OR	95% CI	pval	OR	95% CI	pval
Chemokines																	
	CTACK[c]	3	1.208	(1.037,1.408)	0.015	1.393	(0.968,2.004)	0.325	1.238	(1.049,1.461)	0.011	1.242	(1.001,1.541)	0.187	1.253	(1.035,1.516)	0.147
	Eotaxin[c]	4	0.994	(0.845,1.17)	0.946	1.199	(0.797,1.804)	0.475	1.063	(0.881,1.284)	0.524	1.05	(0.766,1.44)	0.780	1.068	(0.851,1.34)	0.609
	GROa	9	0.961	(0.881,1.047)	0.362	1.043	(0.847,1.284)	0.705	0.936	(0.837,1.047)	0.245	0.929	(0.796,1.084)	0.378	0.974	(0.877,1.081)	0.632
	IP10	9	1.036	(0.898,1.194)	0.631	1.015	(0.697,1.478)	0.940	1.001	(0.81,1.238)	0.993	0.883	(0.605,1.289)	0.537	0.939	(0.692,1.272)	0.694
	MCP1[c]	4	0.980	(0.814,1.18)	0.832	1.142	(0.634,2.057)	0.702	0.894	(0.723,1.105)	0.300	0.919	(0.626,1.349)	0.695	0.881	(0.688,1.129)	0.390
	MCP3[a]	3	0.755	(0.491,1.16)	0.200	0.840	(0.536,1.317)	0.586	0.84	(0.601,1.175)	0.309	1.11	(0.604,2.042)	0.769	0.587	(0.405,0.851)	0.106
	MIG[b]	10	1.002	(0.881,1.138)	0.982	1.118	(0.823,1.52)	0.495	0.987	(0.813,1.197)	0.891	1.08	(0.779,1.499)	0.655	0.955	(0.737,1.237)	0.735
	MIP1a	8	0.996	(0.846,1.172)	0.959	1.247	(0.697,2.231)	0.485	1.041	(0.832,1.301)	0.726	0.982	(0.667,1.446)	0.931	1.038	(0.75,1.437)	0.830
	MIP1b[c]	5	0.943	(0.849,1.048)	0.277	0.855	(0.716,1.021)	0.183	0.914	(0.813,1.028)	0.134	0.906	(0.739,1.112)	0.399	0.916	(0.814,1.032)	0.222
	RANTES	10	1.052	(0.91,1.217)	0.491	1.461	(0.98,2.18)	0.100	1.167	(0.961,1.417)	0.120	1.178	(0.906,1.532)	0.251	1.178	(0.91,1.526)	0.245
	SDF1a	6	1.109	(0.791,1.553)	0.549	1.585	(0.3,8.379)	0.617	0.876	(0.549,1.398)	0.579	0.864	(0.475,1.569)	0.651	0.867	(0.556,1.352)	0.557
Growth factors																	
	bNGF	7	1.057	(0.891,1.255)	0.523	1.088	(0.565,2.098)	0.810	1.09	(0.873,1.362)	0.445	1.095	(0.806,1.488)	0.584	1.098	(0.815,1.48)	0.560
	FGFBasic	4	0.950	(0.661,1.365)	0.783	1.908	(0.156,23.291)	0.663	0.913	(0.569,1.467)	0.708	1.44	(0.653,3.178)	0.433	0.723	(0.371,1.41)	0.411
	GCSF	6	0.872	(0.68,1.119)	0.282	0.964	(0.608,1.527)	0.883	0.907	(0.671,1.225)	0.523	0.872	(0.548,1.388)	0.589	0.918	(0.649,1.297)	0.647
	HGF	7	0.939	(0.761,1.158)	0.555	0.962	(0.608,1.523)	0.876	0.935	(0.71,1.232)	0.634	0.919	(0.628,1.346)	0.681	0.919	(0.646,1.307)	0.656
	MCSF	5	0.958	(0.824,1.113)	0.573	1.189	(0.815,1.733)	0.435	0.911	(0.745,1.114)	0.366	0.793	(0.55,1.144)	0.283	0.788	(0.575,1.079)	0.211
	PDGFbb[c]	3	1.201	(0.987,1.461)	0.067	1.215	(0.849,1.738)	0.480	1.183	(0.965,1.449)	0.105	1.155	(0.876,1.523)	0.415	1.179	(0.942,1.475)	0.287
	SCF	9	1.021	(0.847,1.23)	0.831	0.995	(0.578,1.713)	0.987	1.039	(0.789,1.369)	0.784	1.439	(0.857,2.418)	0.206	1.315	(0.824,2.097)	0.284
	SCGFb[c]	4	0.838	(0.702,1.002)	0.052	0.673	(0.49,0.925)	0.135	0.795	(0.638,0.989)	0.040	0.766	(0.528,1.113)	0.257	0.746	(0.551,1.008)	0.153
	VEGF[c]	3	1.023	(0.934,1.12)	0.630	1.012	(0.763,1.343)	0.947	1.027	(0.935,1.128)	0.582	1.082	(0.905,1.294)	0.477	1.025	(0.935,1.123)	0.650
Interleukins																	
	IL-10	8	1.015	(0.885,1.163)	0.836	1.059	(0.809,1.388)	0.690	1.01	(0.864,1.182)	0.897	0.972	(0.727,1.299)	0.851	1.023	(0.878,1.193)	0.778
	IL-12p70	8	1.046	(0.937,1.168)	0.419	0.994	(0.83,1.191)	0.951	1.026	(0.91,1.158)	0.671	1.091	(0.82,1.452)	0.570	1.027	(0.906,1.164)	0.687
	IL-13	7	1.062	(0.95,1.188)	0.289	0.931	(0.773,1.121)	0.484	1.041	(0.908,1.193)	0.566	1.249	(0.991,1.574)	0.109	1.039	(0.909,1.187)	0.594
	IL-16[c]	3	0.974	(0.873,1.087)	0.637	0.985	(0.741,1.31)	0.934	0.968	(0.86,1.09)	0.593	0.929	(0.771,1.12)	0.521	0.967	(0.857,1.092)	0.647
	IL-17	6	1.369	(1.032,1.816)	0.029	1.058	(0.521,2.148)	0.884	1.441	(1.002,2.073)	0.049	1.037	(0.644,1.67)	0.888	1.419	(0.895,2.25)	0.196
	IL-18	11	1.105	(0.993,1.23)	0.067	1.227	(0.994,1.515)	0.090	1.137	(0.991,1.306)	0.068	1.14	(0.938,1.385)	0.216	1.136	(0.958,1.348)	0.174

	IL1b	6	1.049	(0.831,1.324)	0.686	1.169	(0.707,1.934)	0.576	1.045	(0.778,1.403)	0.772	1.185	(0.799,1.757)	0.436	0.962	(0.665,1.391)	0.844
	IL1ra	7	1.153	(0.951,1.397)	0.147	1.633	(0.943,2.826)	0.140	1.176	(0.915,1.512)	0.206	1.17	(0.78,1.756)	0.477	1.219	(0.841,1.769)	0.336
	IL-2	8	1.168	(0.989,1.378)	0.067	0.985	(0.714,1.359)	0.932	1.089	(0.885,1.34)	0.418	1.083	(0.766,1.532)	0.665	1.041	(0.744,1.457)	0.820
	IL-2ra	6	1.015	(0.89,1.158)	0.821	0.883	(0.692,1.127)	0.373	0.992	(0.852,1.155)	0.918	1.024	(0.735,1.427)	0.892	0.974	(0.834,1.138)	0.754
	IL-4	5	0.951	(0.77,1.174)	0.637	1.127	(0.816,1.558)	0.520	0.975	(0.743,1.278)	0.853	0.875	(0.538,1.424)	0.620	1.053	(0.8,1.387)	0.729
	IL-5	5	0.940	(0.78,1.133)	0.517	0.647	(0.396,1.057)	0.181	0.985	(0.773,1.256)	0.903	0.987	(0.694,1.405)	0.947	1.037	(0.792,1.358)	0.805
	IL-6	4	0.810	(0.605,1.085)	0.158	1.377	(0.811,2.337)	0.358	0.961	(0.657,1.407)	0.840	0.905	(0.507,1.616)	0.759	0.985	(0.653,1.484)	0.946
	IL-7	6	1.049	(0.907,1.213)	0.518	1.146	(0.789,1.665)	0.514	1.072	(0.897,1.282)	0.443	1.114	(0.876,1.416)	0.419	1.082	(0.886,1.322)	0.474
	IL-8[a]	4	1.031	(0.8,1.328)	0.813	1.385	(1.048,1.831)	0.149	0.995	(0.803,1.232)	0.960	0.781	(0.485,1.257)	0.383	1.238	(0.991,1.547)	0.157
	IL-9	4	0.918	(0.722,1.167)	0.483	0.579	(0.313,1.072)	0.224	0.83	(0.609,1.132)	0.240	0.818	(0.527,1.271)	0.438	0.812	(0.567,1.162)	0.337
**Others**																	
	IFNg	4	1.289	(0.924,1.799)	0.135	1.238	(0.639,2.396)	0.592	1.35	(0.911,2.001)	0.135	1.442	(0.794,2.618)	0.316	1.454	(0.871,2.429)	0.248
	MIF	4	1.049	(0.849,1.296)	0.657	0.945	(0.66,1.352)	0.786	1.025	(0.788,1.334)	0.854	1.014	(0.714,1.442)	0.942	1.009	(0.738,1.379)	0.958
	TNFa	4	1.176	(0.961,1.439)	0.115	1.183	(0.811,1.728)	0.475	1.179	(0.926,1.501)	0.183	1.229	(0.914,1.653)	0.266	1.224	(0.908,1.65)	0.277
	TNFb	4	1.055	(0.944,1.179)	0.342	1.132	(0.961,1.333)	0.277	1.088	(0.96,1.233)	0.188	1.052	(0.852,1.3)	0.670	1.099	(0.953,1.267)	0.286
	TRAIL[bc]	4	1.034	(0.882,1.212)	0.678	0.744	(0.401,1.382)	0.449	1.002	(0.817,1.23)	0.981	0.929	(0.635,1.359)	0.729	0.921	(0.738,1.149)	0.520

Abbreviations: CI, Confidence interval; OR, Odds Ratio; pval, p-value; SNPs, single nucleotide polymorphisms

[a]: The MR-Egger (SIMEX) method was applied.

[b]: Outliers detected by MR-PRESSO were removed.

[c]: The cutoff threshold for SNP significance was set as 5×10^{-8}, while for other cytokines was 5×10^{-6}.

OR and 95% CI represent the change in the odds ratio of SLE per 1 SD increase in inflammatory cytokines.

After correcting for multiple comparison, p-value < 0.05/41 = 0.0012 was considered as significant.

Table S2. Heterogeneity and horizontal pleiotropy tests of forty-one inflammatory cytokines on SLE.

Exposures	Q_1 pval	Q_2 pval	I^2	intercept	intercept pval	MR-PRESSO Global Test pval	Recommended Method
bNGF	0.94	0.88	0.0%	-0.004	0.93	0.94	IVW
CTACK	0.67	0.76	0.0%	-0.052	0.55	0.94	IVW
Eotaxin	0.64	0.70	0.0%	-0.045	0.43	0.62	IVW
FGFBasic	0.17	0.11	40.3%	-0.068	0.63	0.20	IVW
GCSF	0.38	0.29	5.7%	-0.016	0.62	0.73	IVW
GROa	0.56	0.53	0.0%	-0.032	0.43	0.63	IVW
HGF	0.62	0.49	0.0%	-0.004	0.91	0.48	IVW
IFNg	0.70	0.49	0.0%	0.008	0.90	0.29	IVW
IL-10	0.56	0.46	0.0%	-0.007	0.73	0.63	IVW
IL-12p70	0.43	0.38	0.0%	0.013	0.50	0.29	IVW
IL-13	0.58	0.90	0.0%	0.042	0.14	0.51	IVW
IL-16	0.24	0.09	30.1%	-0.005	0.94	0.05	IVW
IL-17	0.73	0.70	0.0%	0.044	0.48	0.70	IVW
IL-18	0.84	0.88	0.0%	-0.028	0.29	0.67	IVW
IL1b	0.86	0.79	0.0%	-0.020	0.66	0.85	IVW
IL1ra	0.44	0.53	0.0%	-0.061	0.24	0.29	IVW
IL-2	0.88	0.96	0.0%	0.036	0.27	0.87	IVW
IL-2ra	0.16	0.26	37.4%	0.061	0.23	0.17	IVW
IL-4	0.31	0.41	16.2%	-0.050	0.27	0.30	IVW
IL-5	0.25	0.42	25.8%	0.069	0.21	0.31	IVW
IL-6	0.13	0.98	46.5%	-0.093	0.14	0.20	IVW
IL-7	0.87	0.80	0.0%	-0.029	0.64	0.82	IVW
IL-8	0.07	0.90	56.7%	-0.069	0.12	0.27	IVW
IL-9	0.42	0.88	0.0%	0.108	0.25	0.59	IVW
IP10	0.12	0.08	36.8%	0.004	0.91	0.16	IVW
MCP1	0.40	0.28	0.0%	-0.031	0.64	0.47	IVW
MCP3	0.04	0.02	68.3%	-0.158	0.72	0.26	MR-Egger (SIMEX)
MCSF	0.14	0.21	42.7%	-0.071	0.29	0.27	IVW
MIG	0.11	0.40	37.6%	-0.035	0.43	0.31	IVW
MIF	0.51	0.10	0.0%	0.036	0.55	0.68	IVW
MIP1a	0.14	0.13	35.6%	-0.042	0.45	0.19	IVW
MIP1b	0.43	0.56	0.0%	0.047	0.27	0.55	IVW
PDGFbb	0.79	0.49	0.0%	-0.002	0.95	0.55	IVW
RANTES	0.32	0.50	3.6%	-0.076	0.12	0.33	IVW
SCF	0.08	0.05	43.1%	0.004	0.92	0.05	IVW
SCGFb	0.27	0.54	22.7%	0.070	0.24	0.31	IVW
SDF1a	0.16	0.11	36.8%	-0.036	0.68	0.25	IVW
TNFa	0.96	0.85	0.0%	-0.001	0.97	0.94	IVW
TNFb	0.45	0.50	0.0%	-0.030	0.37	0.47	IVW
TRAIL	0.16	0.20	42.3%	0.227	0.39	0.27	IVW
VEGF	0.24	0.09	29.5%	0.004	0.95	0.55	IVW

Q_1 pval: p value of Q test from IVW method; Q_2 pval: p value of Q test from MR-Egger method

Abbreviations: pval, p-value; Q, Cochran Q statistics; SNPs, single nucleotide polymorphisms; IVW, the inverse variance weighted method; SIMEX, simulation extrapolation.

Table S3. SNPs information of forty-one inflammation cytokines with systemic lupus erythematosus

			inflammatory cytokines				systemic lupus erythematosus		
SNP	effect allele	other allele	Beta	se	pval	F	log(OR)	se	pval
**bNGF**									
rs28637706	G	T	0.1589	0.0263	1.42E-09	36.5	-0.0626	0.2129	0.7686
rs67476890	C	T	-0.1769	0.0379	3.13E-06	21.8	-0.1142	0.2500	0.6478
rs72780728	G	A	-0.1883	0.0403	2.99E-06	21.8	-0.0534	0.3031	0.8602
rs73472576	C	T	0.1181	0.0252	2.69E-06	22.0	0.0851	0.2457	0.7290
rs74966328	G	A	0.2930	0.0618	2.13E-06	22.5	0.2477	0.2363	0.2946
rs7970581	G	T	-0.1380	0.0282	9.27E-07	23.9	0.1435	0.2303	0.5332
rs9436119	G	A	0.1121	0.0246	3.91E-06	20.8	0.0897	0.1884	0.6341
**CTACK**									
rs135564	G	A	0.1893	0.0268	2.43E-12	49.9	0.0531	0.1738	0.7600
rs2070074	G	A	-0.4467	0.0374	1.79E-32	142.7	0.2359	0.1069	0.0273
rs55764737	C	T	-0.5313	0.0972	4.62E-08	29.9	0.1983	0.1513	0.1899
**Eotaxin**									
rs112347425	C	T	-0.1580	0.0277	8.65E-09	32.5	-0.0636	0.2981	0.8311
rs12075	G	A	-0.1671	0.0156	1.33E-26	114.7	-0.1769	0.1631	0.2782
rs2024050	G	A	-0.1728	0.0303	1.10E-08	32.5	0.0576	0.2192	0.7928
rs2228467	C	T	0.4163	0.0292	2.27E-46	203.3	0.0710	0.1153	0.5379
**GROa**									
rs1113500	G	T	-0.1174	0.0244	1.57E-06	23.2	-0.1687	0.2556	0.5093
rs118158560	G	A	-0.2703	0.0594	3.42E-06	20.7	-0.7796	0.3529	0.0271
rs12075	G	A	-0.3751	0.0237	1.24E-55	250.5	-0.0788	0.0727	0.2782
rs140734053	G	A	-0.7257	0.1561	3.58E-06	21.6	0.0540	0.1266	0.6695
rs185768063	G	A	-0.3998	0.0760	1.46E-07	27.7	0.1548	0.3799	0.6838
rs188345231	C	T	-0.6230	0.1323	4.34E-06	22.2	-0.1514	0.2196	0.4906
rs508977	G	T	0.3802	0.0280	7.56E-42	184.4	0.0262	0.0761	0.7310
rs62024303	G	A	0.3053	0.0666	4.41E-06	21.0	-0.0662	0.2869	0.8176
rs78653452	G	T	0.7362	0.1558	1.21E-06	22.3	-0.0663	0.1838	0.7184
**GCSF**									
rs115256310	G	A	0.6821	0.1360	6.73E-07	25.2	-0.0447	0.1930	0.8170
rs11903143	G	A	-0.0870	0.0176	6.35E-07	24.4	-0.4508	0.3336	0.1766
rs1817411	C	T	-0.0890	0.0191	3.10E-06	21.7	0.4407	0.3977	0.2678
rs2671444	G	A	0.0784	0.0166	2.48E-06	22.3	-0.1282	0.3853	0.7394
rs76287671	C	T	-0.0938	0.0189	6.92E-07	24.6	-0.7737	0.4420	0.0800
rs77318030	C	T	0.2045	0.0428	2.21E-06	22.8	-0.1489	0.3567	0.6763
**FGFBasic**									
rs13412535	G	A	0.1112	0.0225	7.35E-07	24.4	-0.5240	0.3587	0.1441
rs747334	G	A	-0.0751	0.0164	4.53E-06	21.0	-0.2690	0.3227	0.4045
rs75168112	C	T	0.1001	0.0214	3.00E-06	21.9	0.4874	0.4254	0.2519
rs9907295	C	T	0.1319	0.0269	7.95E-07	24.0	0.3889	0.3966	0.3269
**HGF**									
rs11060254	G	A	0.0800	0.0167	1.58E-06	22.9	-0.1244	0.2530	0.6230
rs150322232	G	A	-0.2104	0.0463	4.89E-06	20.7	0.5008	0.4239	0.2375
rs1698249	C	A	0.1698	0.0372	4.09E-06	20.8	0.1741	0.2823	0.5374

rs2003620	C	T	-0.2279	0.0489	2.83E-06	21.7	-0.4623	0.2888	0.1095
rs3748034	G	T	-0.1495	0.0234	1.81E-10	40.8	-0.0672	0.4413	0.8789
rs5745687	C	T	0.3072	0.0406	2.75E-14	57.3	-0.0645	0.2007	0.7481
rs62481625	C	T	-0.1091	0.0225	1.18E-06	23.5	-0.0912	0.2953	0.7575
**IFNg**									
rs11843756	G	T	-0.1840	0.0393	3.09E-06	21.9	-0.1076	0.3873	0.7811
rs12420286	C	T	-0.2376	0.0501	2.08E-06	22.5	0.1282	0.4098	0.7544
rs2073438	G	A	-0.0898	0.0188	1.68E-06	22.8	0.4368	0.3974	0.2718
rs78296352	G	T	-0.3430	0.0652	1.38E-07	27.7	0.3820	0.2528	0.1308
**IL10**									
rs10457128	G	A	0.0865	0.0172	5.24E-07	25.3	0.6736	0.3473	0.0524
rs10493718	C	A	0.1100	0.0222	7.16E-07	24.6	-0.2687	0.3154	0.3941
rs2086656	C	T	0.0789	0.0171	3.78E-06	21.3	-0.2561	0.2844	0.3679
rs282258	C	T	-0.0992	0.0162	1.00E-09	37.5	-0.1013	0.2139	0.6358
rs41282660	G	A	0.1194	0.0255	3.72E-06	21.9	0.0833	0.3686	0.8211
rs4349809	G	T	-0.2853	0.0165	5.77E-67	299.0	0.0352	0.0849	0.6782
rs465757	G	A	-0.0840	0.0174	1.17E-06	23.3	0.1196	0.5851	0.8380
rs7088799	G	T	0.0852	0.0167	3.23E-07	26.0	-0.1180	0.5486	0.8298
**IL12p70**									
rs13209117	G	A	-0.1002	0.0186	5.57E-08	29.0	-0.4074	0.3396	0.2303
rs17229494	G	A	0.1172	0.0257	4.93E-06	20.8	0.5773	0.4660	0.2154
rs282258	C	T	-0.0730	0.0156	3.21E-06	21.9	-0.1377	0.2907	0.6358
rs4349809	G	T	-0.3777	0.0159	2.56E-124	564.3	0.0266	0.0641	0.6782
rs71361173	G	T	-0.1110	0.0239	3.06E-06	21.6	0.2744	0.3792	0.4693
rs782107	G	A	-0.0750	0.0156	1.60E-06	23.1	0.5443	0.3643	0.1351
rs79121401	C	T	-0.5548	0.1206	4.24E-06	21.2	-0.0357	0.2447	0.8840
rs9472183	G	A	0.1019	0.0157	8.61E-11	42.1	0.2901	0.2407	0.2281
**IL13**									
rs12623722	G	A	0.1185	0.0258	4.19E-06	21.1	0.3445	0.2930	0.2398
rs139083458	C	T	-0.9902	0.2107	2.81E-06	22.1	-0.0412	0.1134	0.7163
rs27949	C	T	0.1168	0.0252	3.43E-06	21.5	0.2531	0.2367	0.2850
rs6799107	C	T	0.1459	0.0301	1.25E-06	23.5	0.1357	0.2015	0.5006
rs7073807	C	T	-0.1682	0.0356	2.37E-06	22.3	0.3464	0.2638	0.1891
rs75995699	G	A	-0.3319	0.0698	2.64E-06	22.6	-0.2842	0.3493	0.4159
rs9472168	G	A	-0.4244	0.0248	1.08E-65	292.9	0.0476	0.0810	0.5570
**IL16**									
rs1801020	G	A	-0.1733	0.0272	4.53E-10	40.6	0.2263	0.1947	0.2451
rs4253283	C	T	-0.1460	0.0262	1.75E-08	31.1	-0.2086	0.1679	0.2140
rs4778636	G	A	0.7272	0.0633	1.11E-30	132.0	-0.0272	0.0624	0.6624
**IL17**									
rs1530455	C	T	-0.1080	0.0173	4.87E-10	39.0	0.3632	0.3030	0.2307
rs17106604	C	T	-0.1129	0.0225	6.37E-07	25.2	0.6817	0.3568	0.0561
rs17282552	C	T	0.2001	0.0405	8.21E-07	24.4	-0.1522	0.5968	0.7987
rs184080173	C	T	-0.2384	0.0471	4.19E-07	25.6	0.0847	0.2831	0.7647
rs187475560	C	T	0.2434	0.0520	3.29E-06	21.9	-0.0409	0.7129	0.9543
rs78296352	G	T	-0.3027	0.0646	4.27E-06	22.0	0.4329	0.2865	0.1308

**IL18**									
rs10414578	C	T	0.1771	0.0350	4.16E-07	25.6	0.1141	0.2294	0.6190
rs116383510	C	A	0.5426	0.1056	3.00E-07	26.4	0.2089	0.2243	0.3517
rs117266781	C	T	-0.6841	0.1468	3.15E-06	21.7	0.0713	0.1727	0.6797
rs1852105	C	T	-0.3036	0.0661	4.32E-06	21.1	0.1292	0.2187	0.5547
rs1979967	C	T	-0.1402	0.0286	9.45E-07	24.0	-0.1441	0.2027	0.4772
rs2729385	G	A	-0.1231	0.0262	3.79E-06	22.1	0.1609	0.2379	0.4989
rs385076	C	T	0.2432	0.0248	1.66E-22	96.2	0.1252	0.1309	0.3386
rs658805	G	A	-0.1226	0.0244	4.94E-07	25.2	-0.3330	0.2740	0.2243
rs71478720	C	T	0.2669	0.0276	3.71E-22	93.5	0.2318	0.1340	0.0835
rs78623212	C	T	-0.8705	0.1778	6.71E-07	24.0	0.1302	0.1299	0.3163
rs78716465	G	A	-0.3265	0.0682	1.63E-06	22.9	-0.0619	0.2825	0.8266
**IL1b**									
rs115242021	C	A	-0.2326	0.0414	5.07E-07	31.6	0.2505	0.2321	0.2804
rs143319329	C	T	-0.2801	0.0715	2.00E-06	15.3	-0.0721	0.2631	0.7840
rs1942793	G	T	-0.0717	0.0187	4.98E-06	14.7	-0.2818	0.4431	0.5248
rs61335305	C	A	-0.2966	0.0724	1.90E-06	16.8	0.1645	0.4503	0.7149
rs62015704	G	A	-0.1082	0.0283	2.09E-06	14.6	0.1867	0.3615	0.6055
rs9898641	C	T	0.2032	0.0454	3.59E-06	20.0	-0.0495	0.2215	0.8233
**IL1ra**									
rs1054402	C	T	-0.1311	0.0270	1.13E-06	23.6	-0.1541	0.2659	0.5622
rs11627423	C	A	-0.1171	0.0247	2.12E-06	22.5	-0.2524	0.2837	0.3736
rs12121840	C	T	-0.2692	0.0571	2.43E-06	22.2	0.2165	0.2187	0.3222
rs2809154	C	T	0.1786	0.0388	3.74E-06	21.2	0.2872	0.2138	0.1791
rs56134659	G	A	0.1117	0.0237	2.44E-06	22.2	0.0891	0.3115	0.7749
rs61335305	C	A	-0.4453	0.0908	1.00E-06	24.1	0.1096	0.2999	0.7149
rs9623661	C	T	0.1966	0.0426	3.86E-06	21.3	0.5359	0.2734	0.0500
**IL2**									
rs13412535	G	A	-0.1764	0.0332	1.18E-07	28.2	0.3303	0.2261	0.1441
rs170117	C	T	0.1617	0.0349	3.87E-06	21.5	0.3172	0.2293	0.1666
rs2807544	G	A	-0.1175	0.0253	3.41E-06	21.6	0.3338	0.2367	0.1585
rs4634519	G	A	0.1261	0.0269	2.77E-06	22.0	0.0789	0.2929	0.7876
rs61335305	C	A	-0.4514	0.0918	7.32E-07	24.2	0.1081	0.2959	0.7149
rs62124990	G	T	0.6961	0.1495	3.22E-06	21.7	-0.0143	0.1589	0.9283
rs7615304	G	A	0.1172	0.0242	1.21E-06	23.5	0.0858	0.3455	0.8040
rs80336398	C	T	-0.4001	0.0858	2.82E-06	21.7	0.0761	0.2833	0.7881
**IL2ra**									
rs11241559	G	T	0.1264	0.0266	2.00E-06	22.6	0.6597	0.2978	0.0268
rs115360066	G	A	-0.1867	0.0379	8.06E-07	24.3	-0.0533	0.4799	0.9116
rs12722497	C	A	-0.6279	0.0485	1.57E-38	167.6	-0.0322	0.0811	0.6915
rs185231391	C	T	-0.8503	0.1809	1.47E-06	22.1	0.1911	0.1982	0.3350
rs4733117	C	A	-0.1369	0.0292	2.63E-06	22.0	0.2225	0.3462	0.5205
rs61705228	C	T	-0.3303	0.0716	3.99E-06	21.3	-0.2855	0.2245	0.2034
**IL4**									
rs10512267	C	T	0.0824	0.0161	2.94E-07	26.2	-0.1220	0.4593	0.7906
rs116705532	G	T	0.4678	0.0978	1.76E-06	22.9	-0.1096	0.2155	0.6109

rs17713451	G	A	-0.1274	0.0253	4.97E-07	25.4	-0.3204	0.3174	0.3127
rs79597994	C	T	0.5831	0.1270	4.32E-06	21.1	0.1430	0.1525	0.3483
rs9508291	C	T	0.1676	0.0359	3.03E-06	21.8	-0.6286	0.3777	0.0961
**IL5**									
rs11680908	G	A	-0.2634	0.0554	2.03E-06	22.6	-0.4974	0.2291	0.0299
rs6737109	C	T	-0.1160	0.0247	2.40E-06	22.1	0.1742	0.2376	0.4636
rs72831687	G	A	0.5239	0.1109	1.69E-06	22.3	-0.1112	0.2386	0.6411
rs73040130	C	T	-0.2638	0.0529	6.00E-07	24.9	-0.1121	0.2867	0.6960
rs7767396	G	A	-0.1515	0.0246	7.69E-10	37.9	0.0663	0.1512	0.6609
**IL6**									
rs1333040	C	T	-0.0738	0.0158	3.17E-06	21.8	-0.9168	0.3969	0.0209
rs13412535	G	A	0.1164	0.0215	7.34E-08	29.3	-0.5006	0.3427	0.1441
rs73273528	C	T	-0.2672	0.0553	9.58E-07	23.3	-0.0756	0.3325	0.8201
rs76856708	C	T	-0.3289	0.0700	2.61E-06	22.1	0.0614	0.2179	0.7780
**IL7**									
rs17091524	C	T	-0.4924	0.1013	1.91E-06	23.6	0.0829	0.1681	0.6219
rs28793375	C	T	-0.1638	0.0361	4.46E-06	20.6	-0.2492	0.2766	0.3676
rs4320361	G	T	0.3245	0.0249	6.87E-39	169.8	0.0623	0.1104	0.5728
rs75904417	C	A	0.1698	0.0349	1.16E-06	23.7	0.1741	0.2778	0.5309
rs77981494	C	T	0.5178	0.1064	1.07E-06	23.7	0.1307	0.2054	0.5246
rs78346957	G	A	-0.4588	0.1007	4.51E-06	20.8	-0.0890	0.2531	0.7252
**IL8**									
rs11634944	C	T	0.1214	0.0252	1.29E-06	23.2	-0.3363	0.2673	0.2083
rs12075	G	A	-0.1200	0.0236	3.88E-07	25.9	-0.2463	0.2272	0.2782
rs141926526	C	A	0.6149	0.1308	2.57E-06	22.1	0.2131	0.1107	0.0543
rs2673604	C	A	0.1266	0.0255	7.02E-07	24.6	-0.1596	0.2081	0.4432
**IL9**									
rs41294750	C	T	-0.3514	0.0748	2.37E-06	22.1	-0.2065	0.3394	0.5429
rs7232268	G	A	-0.2759	0.0587	2.53E-06	22.1	-0.0732	0.2292	0.7494
rs7242404	G	A	0.1228	0.0264	3.27E-06	21.6	0.3324	0.2923	0.2555
rs76963786	C	T	0.2865	0.0557	4.50E-07	26.5	-0.2362	0.1916	0.2177
**IP10**									
rs10809307	C	T	-0.1305	0.0282	3.64E-06	21.4	-0.3931	0.2279	0.0846
rs113831257	G	A	-0.3592	0.0644	2.53E-08	31.1	-0.1723	0.1988	0.3861
rs11626201	C	A	-0.1162	0.0245	1.93E-06	22.5	-0.0856	0.2376	0.7185
rs143799975	G	A	0.7984	0.1637	1.00E-06	23.8	0.0248	0.1675	0.8823
rs34383175	C	T	0.3153	0.0657	1.51E-06	23.0	-0.2146	0.2275	0.3455
rs7645625	G	T	0.1086	0.0237	4.41E-06	21.0	0.3611	0.2743	0.1879
rs79848609	C	A	-0.2603	0.0537	8.75E-07	23.5	0.4911	0.3046	0.1069
rs8112909	G	A	0.1426	0.0299	1.94E-06	22.7	0.3421	0.2448	0.1623
rs9450351	C	T	0.2768	0.0489	1.48E-08	32.0	0.2444	0.1806	0.1758
**MCP1**									
rs12075	G	A	-0.2185	0.0155	1.44E-44	198.7	-0.1353	0.1248	0.2782
rs2036297	G	A	-0.1190	0.0160	1.09E-13	55.3	-0.2619	0.2737	0.3385
rs2288370	C	T	0.1031	0.0163	2.25E-10	40.0	0.1121	0.1820	0.5379
rs7632755	G	A	-0.2938	0.0316	1.18E-20	86.4	0.1148	0.1636	0.4828

**MCP3**									
rs10892381	C	T	-0.2412	0.0476	3.56E-07	25.7	0.0417	0.2572	0.8713
rs62492260	G	T	0.2788	0.0580	1.54E-06	23.1	-0.5324	0.1593	0.0008
rs73669117	G	A	0.6238	0.1310	2.56E-06	22.7	0.1701	0.3007	0.5716
**MCSF**									
rs12962919	C	T	-0.3052	0.0662	4.65E-06	21.3	-0.2732	0.1635	0.0947
rs56367447	C	T	0.4967	0.0883	1.72E-08	31.6	0.0613	0.1395	0.6601
rs62294910	G	A	-0.3431	0.0691	6.82E-07	24.7	-0.2115	0.2055	0.3034
rs78296352	G	T	-0.5270	0.1112	1.05E-06	22.5	0.2486	0.1646	0.1308
rs9387100	C	T	0.1352	0.0292	4.07E-06	21.4	-0.2186	0.2220	0.3246
**MIF**									
rs118055855	C	T	-0.6907	0.1500	4.13E-06	21.2	0.0146	0.1501	0.9228
rs12594190	G	A	-0.1355	0.0267	3.70E-07	25.8	0.0742	0.2900	0.7981
rs13142904	C	T	0.2230	0.0425	2.56E-07	27.5	0.3739	0.2658	0.1595
rs78098071	C	T	0.4867	0.0918	1.78E-07	28.1	-0.1713	0.2520	0.4966
**MIG**[a]									
rs111607343	G	A	0.5210	0.1119	2.83E-06	21.7	-0.1118	0.1619	0.4897
rs11177248	G	A	-0.3073	0.0670	4.45E-06	21.0	-0.1669	0.2465	0.4984
rs112861654	G	A	0.2765	0.0529	1.81E-07	27.3	0.1418	0.2048	0.4886
rs117831247	C	T	0.8334	0.1754	2.16E-06	22.6	-0.0471	0.1464	0.7479
rs139010077	C	T	-0.4322	0.0950	3.55E-06	20.7	0.0684	0.3395	0.8403
rs1796086	C	T	0.2096	0.0403	2.23E-07	27.1	-0.3978	0.2458	0.1055
rs55876513	G	T	-0.1660	0.0255	8.23E-11	42.4	0.2459	0.1796	0.1710
rs62562991	G	A	-0.6236	0.1260	8.40E-07	24.5	0.3706	0.1836	0.0436
rs77086208	C	T	-0.3226	0.0698	3.83E-06	21.4	2.0770	0.2869	0.0000
rs816960	C	T	0.1224	0.0244	5.01E-07	25.2	0.1216	0.3311	0.7135
**MIP1a**									
rs10835056	G	T	-0.1194	0.0254	2.60E-06	22.1	-0.2551	0.2378	0.2833
rs12690897	G	A	-0.1248	0.0262	2.11E-06	22.7	-0.1619	0.3051	0.5957
rs184154340	G	A	-0.3310	0.0693	1.86E-06	22.8	0.1185	0.2143	0.5803
rs34771762	G	A	-0.2490	0.0523	2.13E-06	22.7	0.3788	0.2743	0.1673
rs57786342	G	A	-0.1314	0.0285	4.06E-06	21.3	0.0757	0.1963	0.6996
rs60198979	G	A	0.2146	0.0458	2.62E-06	22.0	-0.5281	0.2434	0.0300
rs6900267	C	A	0.2429	0.0519	2.89E-06	21.9	0.3548	0.2356	0.1321
rs7232268	G	A	-0.2821	0.0599	2.55E-06	22.2	-0.0716	0.2242	0.7494
**MIP1b**									
rs113010081	C	T	0.5954	0.0236	3.85E-140	636.5	-0.0861	0.0855	0.3136
rs113877493	C	T	0.6124	0.0218	1.62E-173	789.1	-0.0951	0.0791	0.2289
rs117453826	G	A	0.5774	0.0593	5.07E-22	94.8	0.1807	0.2154	0.4014
rs141102180	G	T	-0.3225	0.0393	1.08E-16	67.3	-0.2924	0.3670	0.4256
rs3760440	G	A	-0.1236	0.0162	2.75E-14	58.2	0.2464	0.2212	0.2653
**PDGFbb**									
rs13412535	G	A	-0.3352	0.0214	2.46E-55	245.3	0.1738	0.1190	0.1441
rs2324229	C	T	-0.0894	0.0161	3.48E-08	30.8	0.1124	0.2308	0.6262
rs55680718	C	T	0.1383	0.0246	1.86E-08	31.6	0.3709	0.3062	0.2259
**RANTES**									

rs112072646	G	A	-0.4286	0.0862	6.48E-07	24.7	0.1579	0.1887	0.4028
rs147509526	C	T	0.3580	0.0717	6.93E-07	24.9	0.1728	0.3459	0.6173
rs2251660	C	A	-0.1829	0.0359	3.83E-07	26.0	0.2232	0.2320	0.3360
rs4940620	G	A	0.2494	0.0540	3.54E-06	21.3	0.3086	0.2446	0.2071
rs62438851	G	A	0.1957	0.0414	2.33E-06	22.3	0.1510	0.2298	0.5109
rs7000423	C	T	0.1318	0.0253	1.82E-07	27.1	-0.1533	0.2547	0.5473
rs72793342	G	A	0.1487	0.0308	1.48E-06	23.3	-0.5795	0.2383	0.0150
rs74472919	C	T	-0.3313	0.0605	3.97E-08	30.0	-0.0303	0.2059	0.8829
rs75613039	C	T	-0.3700	0.0810	4.81E-06	20.9	0.0799	0.2592	0.7579
rs818452	C	T	-0.2381	0.0505	2.36E-06	22.2	0.1647	0.2210	0.4561
**SCF**									
rs113127926	C	A	-0.1982	0.0420	2.27E-06	22.3	0.3414	0.3009	0.2565
rs13412535	G	A	0.1067	0.0213	6.04E-07	25.1	-0.5461	0.3739	0.1441
rs1557570	G	T	-0.1186	0.0170	2.74E-12	48.7	0.1670	0.2625	0.5247
rs1568119	C	T	0.5906	0.1129	1.24E-07	27.4	-0.3913	0.2024	0.0532
rs1942355	C	T	0.0716	0.0157	4.70E-06	20.8	-0.2822	0.4692	0.5476
rs4841899	C	T	0.1004	0.0178	1.78E-08	31.8	-0.1001	0.2194	0.6483
rs635634	C	T	0.1032	0.0191	6.74E-08	29.2	0.4970	0.3738	0.1836
rs78666213	G	T	0.2744	0.0576	2.59E-06	22.7	0.3803	0.3369	0.2589
rs80271436	G	A	0.2370	0.0485	9.95E-07	23.9	0.4917	0.2873	0.0870
**SCGFb**									
rs116924815	C	T	-0.6079	0.0738	1.74E-16	67.9	-0.3466	0.1592	0.0294
rs117716477	C	A	-0.8384	0.0841	1.34E-23	99.4	-0.2812	0.1761	0.1104
rs17876031	G	A	0.1514	0.0255	2.25E-09	35.3	-0.0657	0.2184	0.7635
rs4656185	G	A	-0.2050	0.0256	1.16E-15	64.1	0.0966	0.1872	0.6059
**SDF1a**									
rs12407262	G	A	-0.1179	0.0266	3.99E-06	19.6	-0.1714	0.2986	0.5661
rs13400104	G	A	0.0647	0.0189	4.53E-06	11.7	-0.1553	0.9301	0.8674
rs139840550	G	A	-0.1834	0.0549	3.79E-06	11.2	0.8093	0.4151	0.0512
rs4581824	G	T	0.0701	0.0173	3.05E-06	16.4	-0.1434	0.6561	0.8270
rs482700	G	A	0.0893	0.0203	1.57E-06	19.4	-0.1125	0.2929	0.7008
rs67689854	C	A	0.0681	0.0195	3.07E-06	12.2	1.5471	0.7811	0.0476
**TNFa**									
rs10834997	G	A	0.1247	0.0258	1.33E-06	23.4	0.0798	0.2517	0.7512
rs111332265	G	A	0.3766	0.0754	6.63E-07	24.9	0.2044	0.1578	0.1952
rs79105320	G	A	-0.5605	0.1179	3.59E-06	22.6	0.1040	0.2073	0.6160
rs8121916	C	A	-0.1306	0.0278	2.72E-06	22.1	0.2263	0.2571	0.3788
**TNFb**									
rs10925040	C	T	-0.1755	0.0373	2.67E-06	22.1	0.0567	0.1390	0.6834
rs753274	C	T	0.1736	0.0371	2.77E-06	21.9	-0.1755	0.1714	0.3059
rs7629875	G	A	-0.3766	0.0774	1.37E-06	23.7	-0.0526	0.1903	0.7823
rs78296352	G	T	-1.2215	0.1366	4.76E-21	80.0	0.1073	0.0710	0.1308
**TRAIL**[a]									
rs193112415	C	T	1.0421	0.0623	2.15E-62	279.8	-0.0905	0.1172	0.4402
rs57396456	C	T	0.5626	0.0518	1.25E-27	118.0	0.3092	0.1565	0.0482
rs74778900	C	T	-0.5906	0.0532	2.59E-28	123.2	-0.1784	0.2327	0.4433

rs79287178	G	A	0.4317	0.0421	9.12E-25	105.1	0.1188	0.2234	0.5948
**VEGF**									
rs13209117	G	A	-0.1302	0.0201	5.28E-11	42.0	-0.3135	0.2614	0.2303
rs6921438	G	A	0.4900	0.0175	2.09E-171	784.0	0.0205	0.0487	0.6735
rs9472183	G	A	0.1282	0.0170	5.19E-14	56.9	0.2306	0.1913	0.2281

Abbreviations: log(OR): log odds ratio; se: standard error

[a] Outliers detected by MR-PRESSO were removed.

Beta for inflammatory cytokines represent change in standard deviation per 1 copy of effect allele.

log(OR) for SLE represents log(OR) change in SLE risk per 1 copy of effect allele.

Table S4. MR estimates of SLE on forty-one inflammatory cytokines.

			Inverse variance weighted			MR-Egger			Weighted Median			Simple Mode			Weighted Mode		
**Category**	**Outcomes**	**No. of SNPs**	**Beta**	**95% CI**	**pval**	**Beta**	**95% CI**	**pval**	**Beta**	**95% CI**	**pval**	**Beta**	**95% CI**	**pval**	**Beta**	**95% CI**	**pval**
**Chemokines**																	
	CTACK	40	-0.009	(-0.037,0.019)	0.520	0.012	(-0.051,0.074)	0.72	0.012	(-0.031,0.055)	0.58	0.037	(0.13,-0.057)	0.45	0.048	(0.139,-0.044)	0.31
	Eotaxin	40	0.007	(-0.012,0.025)	0.469	0.004	(-0.036,0.044)	0.84	0.014	(-0.013,0.041)	0.32	0.006	(0.055,-0.043)	0.81	0.016	(0.053,-0.02)	0.38
	GROa[a]	39	0.014	(-0.015,0.042)	0.338	-0.021	(-0.087,0.045)	0.53	0.032	(-0.011,0.075)	0.14	-0.033	(0.055,-0.122)	0.47	0.038	(0.101,-0.026)	0.25
	IP10[a]	39	0.040	(0.012,0.067)	0.005	0.042	(-0.024,0.107)	0.22	0.054	(0.009,0.099)	0.02	-0.038	(0.056,-0.132)	0.43	0.041	(0.107,-0.025)	0.23
	MCP1[b]	40	0.020	(-0.002,0.042)	0.078	0.020	(-0.002,0.042)	0.09	0.015	(-0.015,0.046)	0.32	0.058	(0.151,-0.034)	0.22	0.072	(0.167,-0.023)	0.15
	MCP3	38	0.002	(-0.048,0.053)	0.928	-0.095	(-0.218,0.028)	0.14	-0.022	(-0.102,0.058)	0.59	-0.023	(0.048,-0.095)	0.53	-0.003	(0.05,-0.057)	0.91
	MIG[a]	39	0.046	(0.018,0.073)	0.001	0.058	(-0.001,0.118)	0.06	0.054	(0.013,0.095)	0.01	0.048	(0.128,-0.032)	0.24	0.084	(0.157,0.012)	0.03
	MIP1a	40	0.014	(-0.014,0.042)	0.336	-0.006	(-0.068,0.056)	0.85	0.000	(-0.042,0.043)	0.99	0.032	(0.108,-0.043)	0.40	-0.010	(0.038,-0.058)	0.69
	MIP1b	40	0.021	(0.002,0.039)	0.029	-0.015	(-0.059,0.029)	0.51	0.013	(-0.015,0.041)	0.38	0.014	(0.062,-0.035)	0.59	-0.001	(0.036,-0.038)	0.96
	RANTES	40	0.008	(-0.02,0.037)	0.575	-0.010	(-0.072,0.052)	0.75	0.014	(-0.028,0.057)	0.51	0.041	(0.117,-0.034)	0.29	0.031	(0.082,-0.021)	0.25
	SDF1a	40	0.019	(0.00,0.039)	0.045	0.015	(-0.027,0.056)	0.49	0.034	(0.006,0.063)	0.02	0.042	(0.092,-0.008)	0.11	0.038	(0.074,0.001)	0.05
**Growth factors**																	
	bNGF	40	0.035	(0.007,0.063)	0.014	0.005	(-0.062,0.072)	0.88	0.033	(-0.01,0.075)	0.13	0.046	(0.138,-0.045)	0.32	0.038	(0.104,-0.029)	0.28
	FGFBasic	40	0.021	(0.002,0.04)	0.032	0.019	(-0.023,0.06)	0.38	0.028	(0,0.057)	0.05	0.043	(0.091,-0.005)	0.08	0.026	(0.06,-0.008)	0.15
	GCSF	40	0.010	(-0.009,0.028)	0.317	0.047	(0.007,0.088)	0.03	0.032	(0.004,0.06)	0.03	0.034	(0.085,-0.018)	0.21	0.034	(0.063,0.005)	0.03
	HGF	40	0.018	(0,0.037)	0.051	-0.006	(-0.046,0.034)	0.78	0.011	(-0.016,0.038)	0.41	-0.002	(0.051,-0.054)	0.95	-0.006	(0.032,-0.043)	0.76
	MCSF	40	0.011	(-0.022,0.045)	0.513	-0.027	(-0.11,0.056)	0.53	0.011	(-0.041,0.063)	0.67	-0.019	(0.089,-0.126)	0.74	0.026	(0.1,-0.047)	0.49
	PDGFbb	40	-0.003	(-0.021,0.016)	0.764	-0.017	(-0.057,0.023)	0.42	-0.009	(-0.037,0.019)	0.54	0.012	(0.059,-0.035)	0.63	-0.010	(0.023,-0.042)	0.57
	SCF	40	0.014	(-0.005,0.032)	0.147	0.022	(-0.018,0.061)	0.29	0.014	(-0.013,0.04)	0.31	0.003	(0.054,-0.047)	0.89	0.009	(0.048,-0.03)	0.65
	SCGFb	40	0.020	(-0.009,0.048)	0.173	-0.054	(-0.113,0.005)	0.08	0.003	(-0.038,0.043)	0.89	0.003	(0.072,-0.067)	0.94	-0.002	(0.052,-0.057)	0.94
	VEGF	40	-0.006	(-0.026,0.014)	0.573	-0.036	(-0.079,0.007)	0.11	-0.005	(-0.035,0.026)	0.76	0.012	(0.065,-0.04)	0.65	0.000	(0.037,-0.037)	0.98
**Interleukins**																	
	IL10	40	0.010	(-0.009,0.029)	0.317	0.009	(-0.032,0.05)	0.66	0.015	(-0.012,0.043)	0.28	0.023	(0.077,-0.031)	0.41	0.022	(0.057,-0.013)	0.22
	IL12p70	40	0.007	(-0.011,0.026)	0.431	0.005	(-0.035,0.045)	0.81	0.007	(-0.021,0.034)	0.64	0.025	(0.08,-0.03)	0.38	0.027	(0.067,-0.014)	0.20
	IL13	40	0.007	(-0.021,0.035)	0.608	0.002	(-0.062,0.066)	0.95	0.026	(-0.017,0.069)	0.24	0.050	(0.132,-0.032)	0.24	0.032	(0.089,-0.025)	0.28
	IL16	40	0.024	(-0.005,0.052)	0.101	-0.009	(-0.079,0.062)	0.81	0.041	(-0.003,0.085)	0.06	0.061	(0.144,-0.022)	0.16	0.044	(0.105,-0.017)	0.17
	IL17	40	0.016	(-0.003,0.035)	0.104	0.018	(-0.023,0.059)	0.40	0.013	(-0.014,0.039)	0.35	0.018	(0.061,-0.025)	0.42	0.013	(0.045,-0.019)	0.44
	IL18	40	0.013	(-0.015,0.04)	0.369	-0.003	(-0.064,0.059)	0.93	0.016	(-0.024,0.057)	0.42	0.026	(0.101,-0.048)	0.49	0.023	(0.085,-0.04)	0.48
	IL1b	40	0.018	(-0.004,0.04)	0.118	-0.011	(-0.058,0.037)	0.66	0.017	(-0.016,0.049)	0.32	0.042	(0.098,-0.015)	0.16	0.025	(0.066,-0.017)	0.25

	IL1ra	40	0.000	(-0.027,0.028)	0.979	-0.003	(-0.062,0.057)	0.93	0.013	(-0.028,0.054)	0.55	0.011	(0.082,-0.06)	0.76	0.016	(0.071,-0.039)	0.57
	IL2	40	0.004	(-0.024,0.033)	0.771	0.007	(-0.056,0.07)	0.83	-0.008	(-0.049,0.033)	0.70	0.007	(0.079,-0.065)	0.85	-0.005	(0.047,-0.057)	0.85
	IL2ra	40	0.013	(-0.015,0.04)	0.363	-0.029	(-0.097,0.04)	0.41	0.016	(-0.027,0.06)	0.46	0.041	(0.125,-0.043)	0.34	0.034	(0.104,-0.036)	0.34
	IL4	40	0.024	(0.005,0.042)	0.013	0.045	(0.004,0.085)	0.04	0.028	(0,0.055)	0.05	0.015	(0.066,-0.036)	0.56	0.024	(0.059,-0.01)	0.18
	IL5	40	0.020	(-0.009,0.049)	0.172	0.036	(-0.032,0.105)	0.31	0.014	(-0.03,0.058)	0.54	0.005	(0.095,-0.084)	0.91	0.004	(0.061,-0.054)	0.90
	IL6	40	0.019	(0.001,0.038)	0.042	0.043	(0.003,0.083)	0.04	0.020	(-0.007,0.047)	0.15	-0.003	(0.04,-0.047)	0.88	0.017	(0.05,-0.016)	0.33
	IL7	40	0.024	(-0.005,0.052)	0.102	0.017	(-0.045,0.078)	0.59	0.025	(-0.02,0.069)	0.28	0.008	(0.084,-0.067)	0.83	0.023	(0.08,-0.033)	0.42
	IL8	40	0.019	(-0.009,0.048)	0.174	0.016	(-0.045,0.076)	0.61	0.020	(-0.021,0.061)	0.35	0.011	(0.083,-0.06)	0.76	0.014	(0.068,-0.04)	0.60
	IL9	40	0.026	(-0.001,0.054)	0.063	0.028	(-0.037,0.094)	0.40	0.014	(-0.032,0.06)	0.54	0.112	(0.218,0.005)	0.05	-0.019	(0.074,-0.113)	0.69
**Others**																	
	IFNg	40	0.006	(-0.013,0.025)	0.563	0.010	(-0.032,0.051)	0.65	0.019	(-0.01,0.047)	0.20	0.033	(0.084,-0.019)	0.22	0.023	(0.059,-0.014)	0.24
	MIF	40	-0.003	(-0.031,0.025)	0.822	0.006	(-0.055,0.067)	0.85	-0.004	(-0.045,0.036)	0.84	-0.001	(0.069,-0.071)	0.98	0.008	(0.066,-0.049)	0.78
	TNFa	40	0.029	(0,0.057)	0.048	0.024	(-0.037,0.086)	0.44	0.043	(0.005,0.082)	0.03	0.048	(0.112,-0.017)	0.15	0.040	(0.091,-0.01)	0.13
	TNFb	32	0.018	(-0.027,0.063)	0.436	0.045	(-0.054,0.144)	0.38	0.008	(-0.057,0.074)	0.80	-0.007	(0.095,-0.108)	0.90	-0.007	(0.072,-0.085)	0.87
	TRAIL	40	0.007	(-0.012,0.025)	0.464	-0.003	(-0.043,0.038)	0.90	0.005	(-0.022,0.032)	0.72	-0.001	(0.047,-0.05)	0.96	-0.005	(0.029,-0.039)	0.77

Abbreviations: CI, Confidence interval; pval, p-value; SNPs, single nucleotide polymorphisms

[a]: Outliers detected by MR-PRESSO are removed.

[b]: The MR-Egger (SIMEX) method was applied.

Beta and 95% CI represent change in SD of Inflammatory regulators per log odds increase in systemic lupus erythematosus.

After correcting for multiple comparison, p-value < 0.05/41 = 0.0012 was considered as significant.

Table S5. Heterogeneity and horizontal pleiotropy tests of SLE on forty-one inflammatory cytokines.

Cytokine	Q_1 pval	Q_2 pval	I^2	intercept	intercept pval	MR-PRESSO Global Test pval	Recommended Method
bNGF	0.17	0.18	17.2%	0.012	0.32	0.15	IVW
CTACK	0.33	0.31	8.0%	-0.008	0.47	0.31	IVW
Eotaxin	0.53	0.48	0.0%	0.001	0.89	0.56	IVW
FGFBasic	0.74	0.70	0.0%	0.001	0.90	0.74	IVW
GCSF	0.33	0.47	7.7%	-0.015	0.05	0.35	IVW
GROa	0.21	0.23	15.1%	0.014	0.24	0.21	IVW
HGF	0.43	0.47	2.1%	0.009	0.19	0.43	IVW
IFNg	0.62	0.58	0.0%	-0.002	0.84	0.64	IVW
IL10	0.80	0.76	0.0%	0.000	0.98	0.82	IVW
IL12p70	0.63	0.59	0.0%	0.001	0.89	0.62	IVW
IL13	0.29	0.25	10.1%	0.002	0.85	0.30	IVW
IL16	0.08	0.09	24.5%	0.013	0.32	0.10	IVW
IL17	0.90	0.88	0.0%	-0.001	0.91	0.91	IVW
IL18	0.41	0.38	3.5%	0.006	0.58	0.36	IVW
IL1b	0.67	0.70	0.0%	0.011	0.20	0.66	IVW
IL1ra	0.51	0.46	0.0%	0.001	0.91	0.52	IVW
IL2	0.41	0.37	3.3%	-0.001	0.92	0.42	IVW
IL2ra	0.07	0.09	26.1%	0.016	0.19	0.06	IVW
IL4	0.47	0.49	0.0%	-0.008	0.26	0.50	IVW
IL5	0.16	0.15	17.9%	-0.006	0.60	0.18	IVW
IL6	0.53	0.56	0.0%	-0.009	0.19	0.57	IVW
IL7	0.58	0.54	0.0%	0.003	0.80	0.58	IVW
IL8	0.84	0.81	0.0%	0.001	0.89	0.88	IVW
IL9	0.22	0.19	14.4%	-0.001	0.95	0.17	IVW
IP10	0.25	0.21	12.8%	-0.001	0.95	0.21	IVW
MCP1	0.04	0.04	29.4%	-0.006	0.51	0.04	MR-Egger (SIMEX)
MCP3	0.08	0.13	25.4%	0.038	0.09	0.08	IVW
MCSF	0.10	0.10	23.3%	0.015	0.32	0.10	IVW
MIF	0.91	0.90	0.0%	-0.004	0.74	0.91	IVW
MIG	0.48	0.44	0.0%	-0.005	0.64	0.45	IVW
MIP1a	0.41	0.39	3.1%	0.008	0.48	0.41	IVW
MIP1b	0.10	0.16	23.0%	0.014	0.08	0.11	IVW
PDGFbb	0.67	0.66	0.0%	0.005	0.44	0.69	IVW
RANTES	0.47	0.45	0.0%	0.007	0.51	0.49	IVW
SCF	0.90	0.88	0.0%	-0.003	0.66	0.89	IVW
SCGFb	0.39	0.69	4.3%	0.029	0.01	0.37	IVW
SDF1a	0.70	0.66	0.0%	0.002	0.79	0.72	IVW
TNFa	0.94	0.93	0.0%	0.002	0.88	0.96	IVW
TNFb	0.71	0.68	0.0%	-0.010	0.55	0.73	IVW
TRAIL	0.45	0.42	1.1%	0.004	0.60	0.45	IVW
VEGF	0.48	0.54	0.0%	0.012	0.13	0.49	IVW

Q_1 pval: p value of Q test from IVW method; Q_2 pval: p value of Q test from MR-Egger method

Abbreviations: pval, p-value; Q, Cochran Q statistics; SNPs, single nucleotide polymorphisms; IVW, the inverse variance weighted method; SIMEX, simulation extrapolation.

Table S6. Details of systemic lupus erythematosus predicting SNPs with inflammatory cytokines

SNP	effect allele	other allele	chrom	EAF	F	log(OR)	pval	se
rs10048743	T	G	2	0.859	31.46	0.587	2.04E-08	0.041
rs10200680	T	C	2	0.144	34.20	0.564	4.96E-09	0.042
rs1078324	A	C	5	0.050	83.28	0.193	7.11E-20	0.078
rs10912578	G	A	1	0.699	63.45	0.566	1.65E-15	0.031
rs1143679	A	G	16	0.131	212.00	3.821	5.03E-48	0.040
rs12094036	C	T	1	0.082	32.24	0.469	1.37E-08	0.058
rs12524498	T	G	6	0.011	31.07	0.212	2.48E-08	0.121
rs13019891	T	G	2	0.451	374.85	0.274	1.65E-83	0.029
rs13136219	T	C	4	0.380	39.37	0.669	3.50E-10	0.028
rs13332649	G	A	16	0.198	70.17	0.484	5.43E-17	0.038
rs143123127	A	G	17	0.031	31.28	2.951	2.23E-08	0.084
rs1464446	T	G	3	0.179	66.94	0.469	2.79E-16	0.040
rs17849501	T	C	1	0.060	264.48	6.470	1.81E-59	0.050
rs2431697	C	T	5	0.431	58.01	0.598	2.60E-14	0.029
rs2459611	T	C	2	0.875	33.37	1.825	7.62E-09	0.045
rs2573219	C	A	2	0.087	187.47	3.871	1.13E-42	0.043
rs268124	T	C	2	0.726	33.13	1.536	8.60E-09	0.032
rs34703115	C	T	2	0.033	34.58	0.242	4.08E-09	0.105
rs35000415	T	C	7	0.100	200.23	3.871	1.86E-45	0.042
rs35251378	A	G	19	0.269	52.84	0.581	3.61E-13	0.032
rs353608	G	A	11	0.548	44.22	1.536	2.93E-11	0.028
rs3747093	A	G	22	0.202	57.81	1.830	2.88E-14	0.035
rs389884	G	A	6	0.074	460.99	8.477	2.93E-102	0.043
rs4274624	T	C	2	0.768	293.25	0.276	9.73E-66	0.033
rs4388254	T	C	5	0.071	39.26	2.390	3.71E-10	0.060
rs4661543	G	T	1	0.873	41.94	1.881	9.40E-11	0.042
rs4916215	T	C	1	0.746	43.15	1.672	5.07E-11	0.034
rs57844307	A	G	6	0.082	29.73	1.895	4.97E-08	0.051
rs58688157	G	A	11	0.268	44.20	0.598	2.97E-11	0.034
rs58721818	T	C	6	0.025	75.66	4.545	3.38E-18	0.076
rs597808	G	A	12	0.534	30.40	0.688	3.51E-08	0.029
rs6671847	A	G	1	0.487	47.13	1.581	6.64E-12	0.029
rs6679677	A	C	1	0.092	52.39	2.170	4.55E-13	0.046
rs6889239	C	T	5	0.258	76.51	1.895	2.19E-18	0.032
rs7097397	A	G	10	0.396	42.12	0.651	8.60E-11	0.029
rs73068668	A	G	19	0.095	29.97	0.484	4.40E-08	0.057
rs7768653	T	C	6	0.598	48.62	0.621	3.11E-12	0.030
rs7823055	T	G	8	0.577	150.11	0.446	1.64E-34	0.029
rs7899626	T	C	10	0.363	30.06	1.522	4.19E-08	0.033
rs9852014	G	A	3	0.075	158.63	4.174	2.26E-36	0.049
rs115751548[a]	T	G	6	0.012	33.68	0.193	6.507E-09	0.123

Abbreviations: EAF: effect allele frequency, log(OR): log odds ratio, se: standard error; chrom: chromosome
log(OR) for SLE represents log(OR) change in SLE risk per 1 copy of effect allele
[a]: The proxy SNP was included as the substitute for the missing rs12524498 data in VEGF GWAS.

Fig S1. Forest plots and leave-one-out sensitivity analyses of Mendelian randomization analyses for CTACK and IL-17 in SLE.

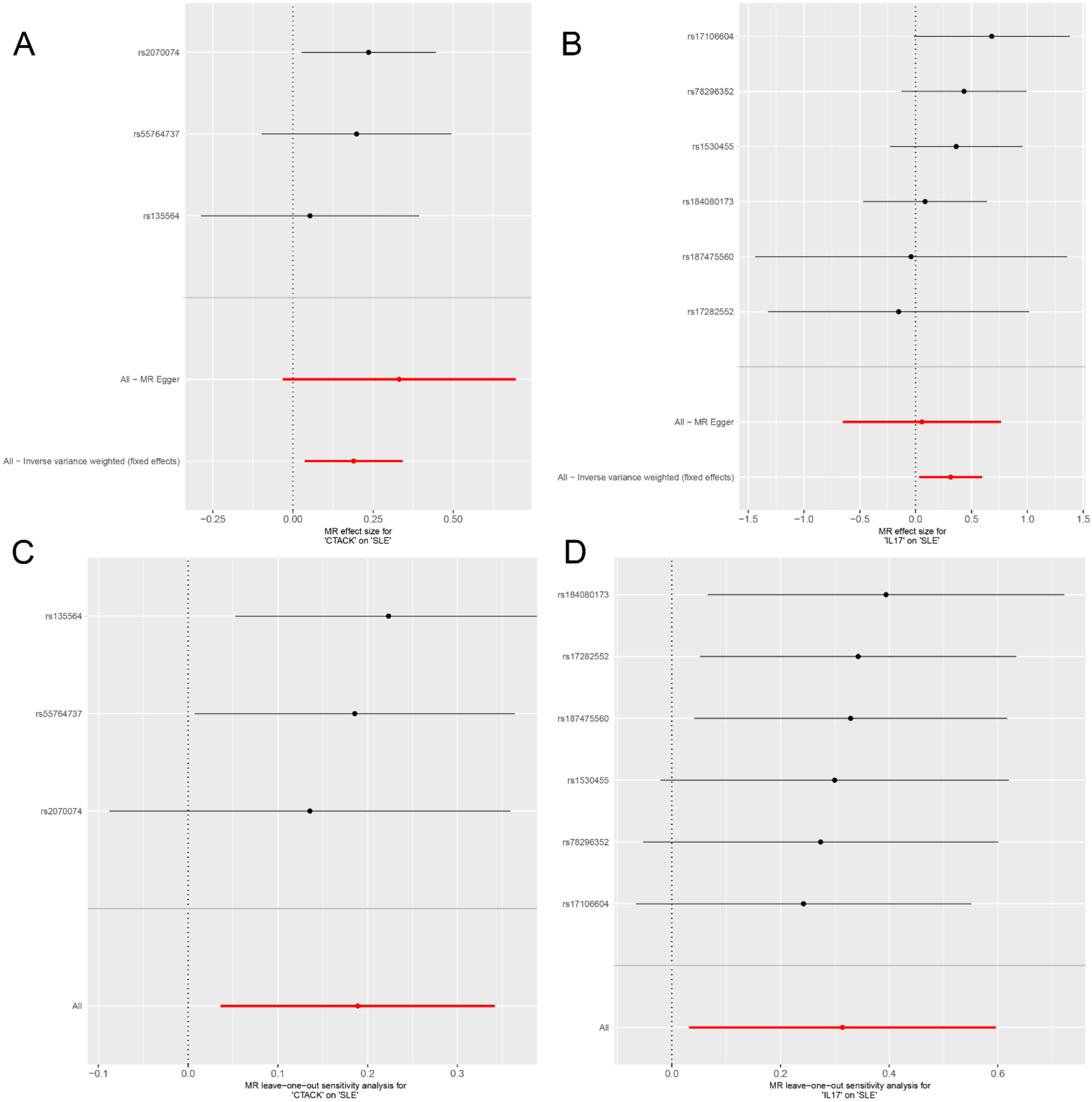

Fig S2. Forest plots of Mendelian randomization analyses between SLE and inflammatory cytokines. (A-I): bNGF, FGFbasic, IL-4, IL-6, IP10, MIG, MIP1b, SDF1a, and TNFa.

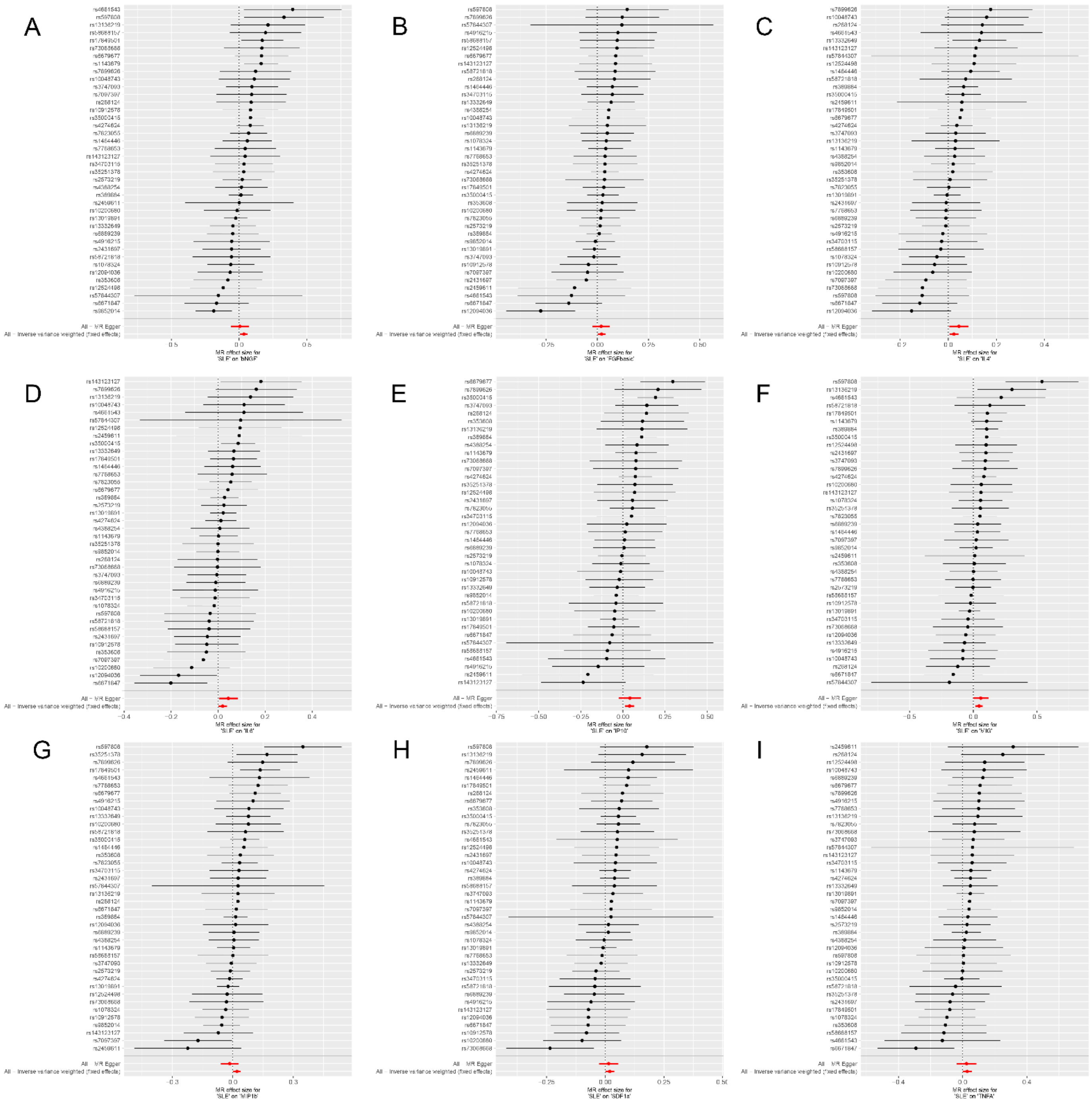

Fig S3. Leave-one-out sensitivity analyses of Mendelian randomization analyses between SLE and inflammatory cytokines. (A-I): bNGF, FGFbasic, IL-4, IL-6, IP10, MIG, MIP1b, SDF1a, and TNFa.

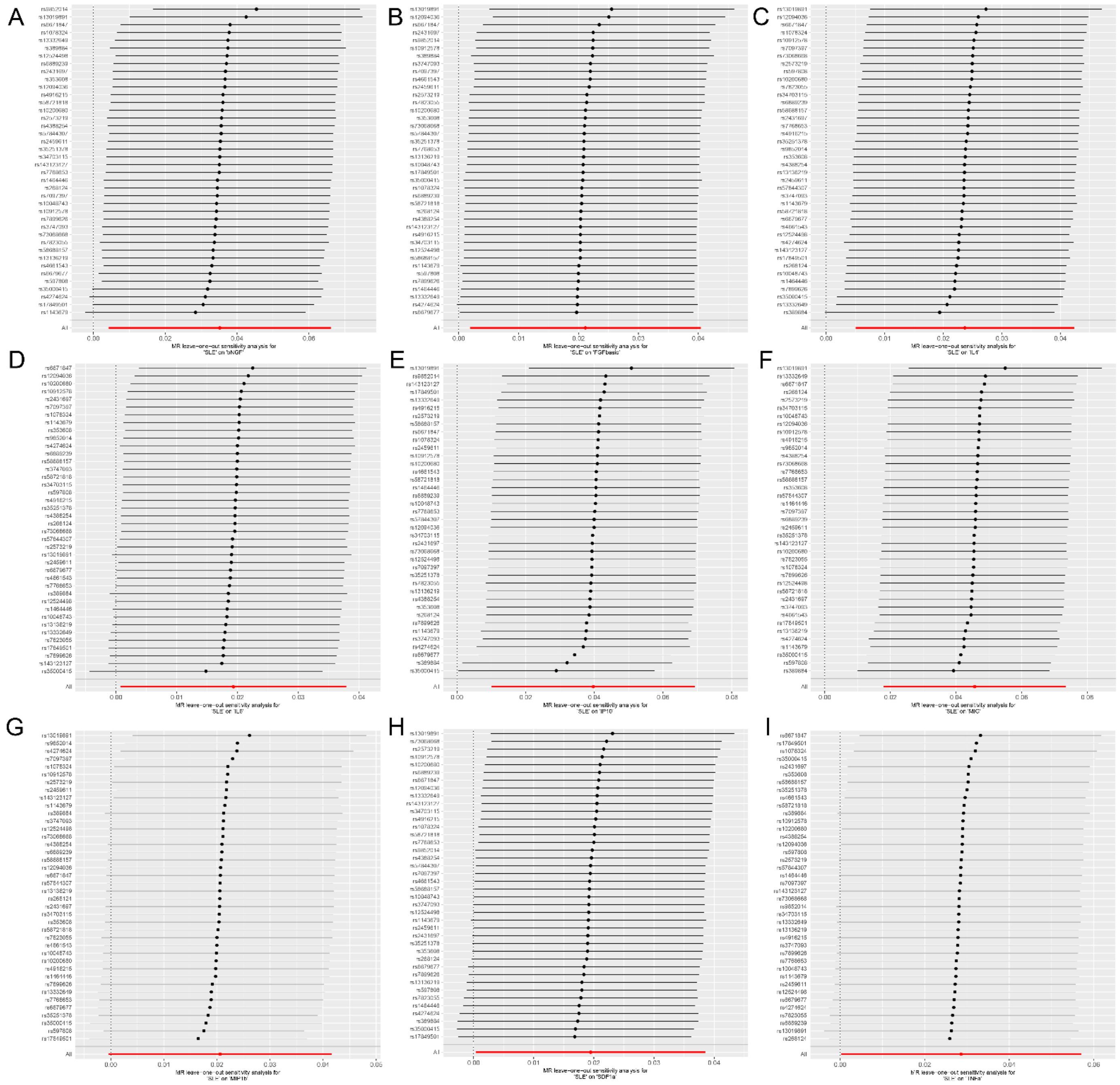

Table S7. STROBE-MR checklist of recommended items to address in reports of Mendelian randomization studies

Item No.	Section	Checklist item	Page No.	Relevant text from manuscript
1	**TITLE and ABSTRACT**	Indicate Mendelian randomization (MR) as the study's design in the title and/or the abstract if that is a main purpose of the study	1	Exploring Causal Correlations Between Inflammatory Cytokines and Systemic Lupus Erythematosus: A Mendelian Randomization
	**INTRODUCTION**			
2	**Background**	Explain the scientific background and rationale for the reported study. What is the exposure? Is a potential causal relationship between exposure and outcome plausible? Justify why MR is a helpful method to address the study question	3	Mendelian randomization (MR) is known as the analytic approach……enhancing the test's applicability and efficacy.
3	**Objectives**	State specific objectives clearly, including pre-specified causal hypotheses (if any). State that MR is a method that, under specific assumptions, intends to estimate causal effects	3	In this study, we first extracted …… the direction of causation was further explored by reversing the exposure and outcome.
	**METHODS**			
4	**Study design and data sources**	Present key elements of the study design early in the article. Consider including a table listing sources of data for all phases of the study. For each data source contributing to the analysis, describe the following:		
	a)	Setting: Describe the study design and the underlying population, if possible. Describe the setting, locations, and relevant dates, including periods of recruitment, exposure, follow-up, and data collection, when available.	4	Two datasets used ……Young Finns Study (YFS) and FINRISK surveys.
	b)	Participants: Give the eligibility criteria, and the sources and methods of selection of participants. Report the sample size, and whether any power or sample size calculations were carried out prior to the main analysis	4	Two datasets used ……Young Finns Study (YFS) and FINRISK surveys.
	c)	Describe measurement, quality control and selection of genetic variants	4	First, we set $p<5\times10^{-8}$ as with proxy SNPs ($R^2>0.9$) …… from LDlink (https://ldlink.nci.nih.gov/)
	d)	For each exposure, outcome, and other relevant variables, describe methods of assessment and diagnostic criteria for diseases	4	The SLE patients were all diagnosed …… Rheumatology (ACR) classification criteria.
	e)	Provide details of ethics committee approval and participant informed consent, if relevant	3	The ethics committees at each institutional …… approval or informed consent was required.
5	**Assumptions**	Explicitly state the three core IV assumptions for the main analysis (relevance, independence and exclusion restriction) as well assumptions for any additional or sensitivity analysis	3	There are three core assumptions……Then, the potential causal interactions were explored (Figure 1).
6	**Statistical methods: main analysis**	Describe statistical methods and statistics used		
	a)	Describe how quantitative variables were handled in the analyses (i.e., scale, units, model)	4-5	The causal association could be evaluated……measure heterogeneity among the estimates from each SNP.
	b)	Describe how genetic variants were handled in the analyses and, if applicable, how their weights were selected	4	First, we set $p<5\times10^{-8}$ as the genome-wide significant threshold……was estimated using the F-statistic to avoid weak instrument bias.
	c)	Describe the MR estimator (e.g. two-stage least squares, Wald ratio) and related statistics. Detail the included	4-5	The causal association could be evaluated……measure

		covariates and, in case of two-sample MR, whether the same covariate set was used for adjustment in the two samples		heterogeneity among the estimates from each SNP.
	d)	Explain how missing data were addressed	5	If the SNP as IV contain missing data in the exposure or outcome summary, it would be omitted.
	e)	If applicable, indicate how multiple testing was addressed	5	The major assessment for each regulator among……were carried out concurrently using other analytical techniques.
7	**Assessment of assumptions**	Describe any methods or prior knowledge used to assess the assumptions or justify their validity	5	For IV-exposure relevance ascertainment, F statistic….. were carried out concurrently using other analytical techniques.
8	**Sensitivity analyses and additional analyses**	Describe any sensitivity analyses or additional analyses performed (e.g. comparison of effect estimates from different approaches, independent replication, bias analytic techniques, validation of instruments, simulations)	4	Weighted Median method has a……among the estimates from each SNP.
9	**Software and pre-registration**			
	a)	Name statistical software and package(s), including version and settings used	5	TwoSample(22) MR package and MR-PRESSO(19) in R (version 4.1.2) were used to conduct the analysis.
	b)	State whether the study protocol and details were pre-registered (as well as when and where)	5	The study weren't pre-registered at any platforms.
	**RESULTS**			
10	**Descriptive data**			
	a)	Report the numbers of individuals at each stage of included studies and reasons for exclusion. Consider use of a flow diagram	4	The one of SLE was obtained from a meta-analysis study comprising 7,219 cases…in 8293 Finnish individuals.
	b)	Report summary statistics for phenotypic exposure(s), outcome(s), and other relevant variables (e.g. means, SDs, proportions)	4	Two datasets used…and outcome group.
	c)	If the data sources include meta-analyses of previous studies, provide the assessments of heterogeneity across these studies	Not available	Not provided in original research
	d)	For two-sample MR:<br>i. Provide justification of the similarity of the genetic variant-exposure associations between the exposure and outcome samples<br>ii. Provide information on the number of individuals who overlap between the exposure and outcome studies	4	There would be no overlap in population selection between exposure group and outcome group.
11	**Main results**			
	a)	Report the associations between genetic variant and exposure, and between genetic variant and outcome, preferably on an interpretable scale	5-6	Nine out of forty-one accessible systemic inflammatory regulators…that weak instrument bias is unlikely to be significant (Table S1-S3).<br>Forty significant SNPs were extracted…The median value of F

					statistic was 47.87 (range 29.73-461).
		b)	Report MR estimates of the relationship between exposure and outcome, and the measures of uncertainty from the MR analysis, on an interpretable scale, such as odds ratio or relative risk per SD difference	5-6	It was found by the IVW method that…in IL-17 with SLE (IVW: OR: 1.37, 95%CI: 1.03–1.82, p=0.029; Weighted Median: OR: 1.44, 95%CI: 1.00-2.07, p=0.049).<br>The findings of IVW method's revealed…tumor necrosis factor alpha (TNFa) (Beta: 0.029, 95%CI: 0-0.057, p=0.048).
		c)	If relevant, consider translating estimates of relative risk into absolute risk for a meaningful time period	No relavant	
		d)	Consider plots to visualize results (e.g. forest plot, scatterplot of associations between genetic variants and outcome versus between genetic variants and exposure)	Figure 2-6	
12	**Assessment of assumptions**				
		a)	Report the assessment of the validity of the assumptions	5-6 Table S2&S5	The median value of F statistic was 47.87 (range 29.73-461).<br>Variance explained by the SNPs …bias is unlikely to be significant (Table S1-S3).
		b)	Report any additional statistics (e.g., assessments of heterogeneity across genetic variants, such as I^2, Q statistic or E-value)	Table S2&S5	
13	**Sensitivity analyses and additional analyses**				
		a)	Report any sensitivity analyses to assess the robustness of the main results to violations of the assumptions	6 Table S1&S4	MR Egger analysis failed to detect…in IL-17 with SLE (IVW: OR: 1.37, 95%CI: 1.03–1.82, p=0.029; Weighted Median: OR: 1.44, 95%CI: 1.00-2.07, p=0.049).<br>The weighted median estimator provided…all suggestively significant regulators were presented in Figure S2-S3.
		b)	Report results from other sensitivity analyses or additional analyses	6 Table S1&S4	MR Egger analysis failed to detect…in IL-17 with SLE (IVW: OR: 1.37, 95%CI: 1.03–1.82, p=0.029; Weighted Median: OR: 1.44, 95%CI: 1.00-2.07, p=0.049).<br>The weighted median estimator provided…all suggestively significant regulators were presented in Figure S2-S3.
		c)	Report any assessment of direction of causal relationship (e.g., bidirectional MR)	5-6	There were no reverse causalities found between a single biomarker and SLE.
		d)	When relevant, report and compare with estimates from non-MR analyses	No	

				relevant	
		e)	Consider additional plots to visualize results (e.g., leave-one-out analyses)	Figure S1-S3	
	**DISCUSSION**				
14	**Key results**		Summarize key results with reference to study objectives	7	In this two-sample MR analysis…are more likely to lie downstream during the disease progression.
15	**Limitations**		Discuss limitations of the study, taking into account the validity of the IV assumptions, other sources of potential bias, and imprecision. Discuss both direction and magnitude of any potential bias and any efforts to address them	8	However, there are several limitations that…so it should be cautious if the conclusions would be applied in other races.
16	**Interpretation**				
		a)	Meaning: Give a cautious overall interpretation of results in the context of their limitations and in comparison with other studies	7	Until now, extensive research has been published…which could not be distinguished by ordinary observational studies.
		b)	Mechanism: Discuss underlying biological mechanisms that could drive a potential causal relationship between the investigated exposure and the outcome, and whether the gene-environment equivalence assumption is reasonable. Use causal language carefully, clarifying that IV estimates may provide causal effects only under certain assumptions	7-8	CTACK is a kind of skin-specific chemokines…chain of events in the disease context remains as meaningful questions to be solved.
		c)	Clinical relevance: Discuss whether the results have clinical or public policy relevance, and to what extent they inform effect sizes of possible interventions	8	Additionally, several exploratory trials…their therapeutic utility may be more plausible.
17	**Generalizability**		Discuss the generalizability of the study results (a) to other populations, (b) across other exposure periods/timings, and (c) across other levels of exposure	8	Second, the data for…so it should be cautious if the conclusions would be applied in other races
	**OTHER INFORMATION**				
18	**Funding**		Describe sources of funding and the role of funders in the present study and, if applicable, sources of funding for the databases and original study or studies on which the present study is based	9	This research was financially … Key Clinical Specialty [No-shslczdzk01002].
19	**Data and data sharing**		Provide the data used to perform all analyses or report where and how the data can be accessed, and reference these sources in the article. Provide the statistical code needed to reproduce the results in the article, or report whether the code is publicly accessible and if so, where	9	All relevant data in this study were uploaded in the article and supplementary materials.
20	**Conflicts of Interest**		All authors should declare all potential conflicts of interest	9	The authors declare that … construed as a potential conflict of interest.

This checklist is copyrighted by the Equator Network under the Creative Commons Attribution 3.0 Unported (CC BY 3.0) license.
